# Sulforaphane protects developing neural networks from VPA-induced synaptic alterations

**DOI:** 10.1038/s41380-025-02967-5

**Published:** 2025-04-02

**Authors:** Riley N. Bessetti, Michelle Cobb, Rosario M. Lilley, Noah Z. Johnson, Daisy A. Perez, Virginia M. Koonce, Krista McCoy, Karen A. Litwa

**Affiliations:** 1 Department of Anatomy and Cell Biology, Brody School of Medicine, East Carolina University (ECU), Greenville, NC USA; 2 East Carolina Diabetes and Obesity Institute at ECU, Greenville, NC USA; 3 Florida Oceanographic Society, Stuart, FL USA

**Keywords:** Autism spectrum disorders, Cell biology, Stem cells, Neuroscience

## Abstract

Prenatal brain development is particularly sensitive to chemicals that can disrupt synapse formation and cause neurodevelopmental disorders. In most cases, such chemicals increase cellular oxidative stress. For example, prenatal exposure to the anti-epileptic drug valproic acid (VPA), induces oxidative stress and synaptic alterations, promoting autism spectrum disorders (ASD) in humans and autism-like behaviors in rodents. Using VPA to model chemically induced ASD, we tested whether activation of cellular mechanisms that increase antioxidant gene expression would be sufficient to prevent VPA-induced synaptic alterations. As a master regulator of cellular defense pathways, the transcription factor nuclear factor erythroid 2-related factor 2 (NRF2) promotes expression of detoxification enzymes and antioxidant gene products. To increase NRF2 activity, we used the phytochemical and potent NRF2 activator, sulforaphane (SFN). In our models of human neurodevelopment, SFN activated NRF2, increasing expression of antioxidant genes and preventing oxidative stress. SFN also enhanced expression of genes associated with synapse formation. Consistent with these gene expression profiles, SFN protected developing neural networks from VPA-induced reductions in synapse formation. Furthermore, in mouse cortical neurons, SFN rescued VPA-induced reductions in neural activity. These results demonstrate the ability of SFN to protect developing neural networks during the vulnerable period of synapse formation, while also identifying molecular signatures of SFN-mediated neuroprotection that could be relevant for combatting other environmental toxicants.

## Introduction

The past decades have witnessed increasing awareness of the contribution of environmental risk factors to neurodevelopmental disorders, particularly autism spectrum disorders (ASDs) [1–4]. These environmental risk factors include pharmaceuticals, maternal infection, and chemical pollutants [1–4]. In many cases, these risk factors promote cellular oxidative stress, during which production of reactive oxygen species (ROS) outpaces the ability of cellular antioxidant defense mechanisms to restore physiological ROS levels [5, 6]. In the brain, this delicate equilibrium between ROS and antioxidant activity is critical, as ROS exhibit both beneficial and detrimental effects. For example, ROS guide neurite outgrowth and regulate synaptic plasticity [7–10]. However, elevated ROS levels can disrupt these physiological processes, resulting in cognitive decline [9–11]. Prolonged ROS elevation results in damage to proteins, lipids, and nucleotides, leading to accumulation of byproducts [e.g., 3-nitrotyrosine [12, 13], 8-iso-prostaglandin F_2α_ [14], and 8-oxo-2′-deoxyguanine [15]] that adversely impact macromolecular function. These oxidative stress byproducts are emerging biomarkers for ASD [5, 16–21]. Thus, while there are positive roles for ROS in brain development, many environmental factors promote excessive ROS production that threatens the developing brain. Furthermore, the ever-growing list of chemical factors linked to neurodevelopmental disorders impossibly limits our ability to eliminate all risk factors. Preventative strategies are needed to combat the adverse effects of environmental pollutants on neurodevelopment. To address this need, we tested whether upregulation of the body’s innate Nuclear Factor E2-related Factor 2 (NRF2) cellular antioxidant defense mechanisms could prevent neurodevelopmental alterations in a model of chemically induced ASD.

NRF2 protects cells from a variety of external insults by acting as a first responder to alleviate oxidative stress [22]. Under basal conditions, the ubiquitin ligase, Kelch-like ECH-associated protein 1 (KEAP1) sequesters NRF2 in the cytosol and targets it for proteasomal degradation [22, 23]. However, the KEAP1-NRF2 interaction also facilitates rapid NRF2 activation under conditions of oxidative stress [22, 23]. In response to oxidative stress, cysteine residues within KEAP1 become oxidized and release NRF2 [22, 24]. Once released, NRF2 is phosphorylated and translocates to the nucleus [22]. As a transcription factor, NRF2 binds to specific enhancer sequences termed antioxidant response elements (AREs) to promote transcription of numerous detoxification enzymes and antioxidant genes [25]. Thus, NRF2 activates pathways that reduce the toxic insult and restore physiological ROS levels. In addition to oxidative stress, electrophilic phytochemicals alkylate KEAP1 cysteine residues to potently induce NRF2 nuclear translocation and transcriptional activity [26]. Of all such phytochemicals, sulforaphane (SFN) is the most potent NRF2 activator [27]. Electrophilic phytochemicals, such as SFN, modify distinct cysteine residues from those affected by oxidative stress [24], allowing the phytochemicals to further upregulate NRF2 activity and mitigate adverse outcomes of external insults [28, 29].

SFN is abundant in the sprouts of cruciferous vegetables, such as broccoli [30]. Upon damage or insult to the plant, the enzyme myrosinase hydrolyzes the precursor glucoraphanin to produce SFN [30]. Notably, SFN is significantly higher in sprouts (10–100×) than the mature plant, suggesting that SFN protects the plant during sensitive developmental periods [31, 32]. Through its ability to activate NRF2, SFN’s beneficial properties extend to mammalian cells and protect them from various external insults, including toxicants such as the endocrine disrupting chemical vinclozolin [33], nutrient deprivation [34, 35], and hypoxia [28, 36]. SFN shows promise for treatment or prevention of diverse disorders including hypospadias [33], pre-eclampsia [37, 38], and dementia [30, 39–42]. SFN may also reduce the symptoms of ASD in adolescents [43, 44]. Importantly for our studies, sulforaphane is lipophilic and able to cross the blood-brain barrier [45]. However, despite extensive research on SFN’s beneficial properties, its implications for brain health are limited to adolescents and adults [46, 47]. Furthermore, prior research focused on treating symptoms of neurological disorders, rather than preventing their occurrence. We currently lack research on whether SFN protects the developing fetal brain, which is particularly susceptible to external insults. Given that chemicals and other environmental factors strongly influence the risk of ASD development [1, 2, 48], we tested whether SFN could prevent synaptic alterations in a chemically induced ASD model.

To elucidate SFN’s neuroprotective effects, we used the antiepileptic drug valproic acid (VPA) to model ASD. Prenatal VPA exposure increases the likelihood of human offspring developing ASD and similarly leads to autism-like behaviors in rodents [49–51]. Importantly, VPA increases cellular oxidative stress [52–54], similar to other scenarios in which SFN has beneficial properties [55]. While VPA increases oxidative stress, in several systems, it suppresses NRF2 activity [56–58]. In cellular models of human brain development, VPA exposure increased expression of ASD-associated genes [59–61]. Using models of human brain development, we researched the early implications of VPA exposure on developing neural networks and demonstrated SFN’s ability to combat VPA-induced oxidative stress. To uncover molecular signatures underlying VPA resistance, we used RNA sequencing (RNA-seq) technology. Interestingly, low dose SFN (0.1 μM) alone minimally impacted gene expression signatures. However, in combination with VPA, SFN significantly upregulated expression of genes associated with NRF2 transcription and synapse formation. These transcriptomic changes correlated with synaptic resiliency in the presence of VPA-induced synapse loss. SFN protected synapse loss in both human-derived cortical neurons and primary mouse cortical neurons. In contrast to human-derived cortical spheroids which exhibited highly variable immature neural activity, mouse cortical neurons developed more synchronous activity. In mouse cortical neurons, VPA reduced neural activity, which was rescued in the presence of SFN. These results demonstrate that SFN successfully combats early events in VPA-induced neural network changes. This study also identifies key molecular pathways underlying SFN-mediated neuroprotection, paving the way for future research to examine SFN’s ability to protect vulnerable synapses and successfully combat other environmental risk factors that threaten brain development.

## Results

### Sulforaphane promotes NRF2 antioxidant activity

**Fig. 1:**
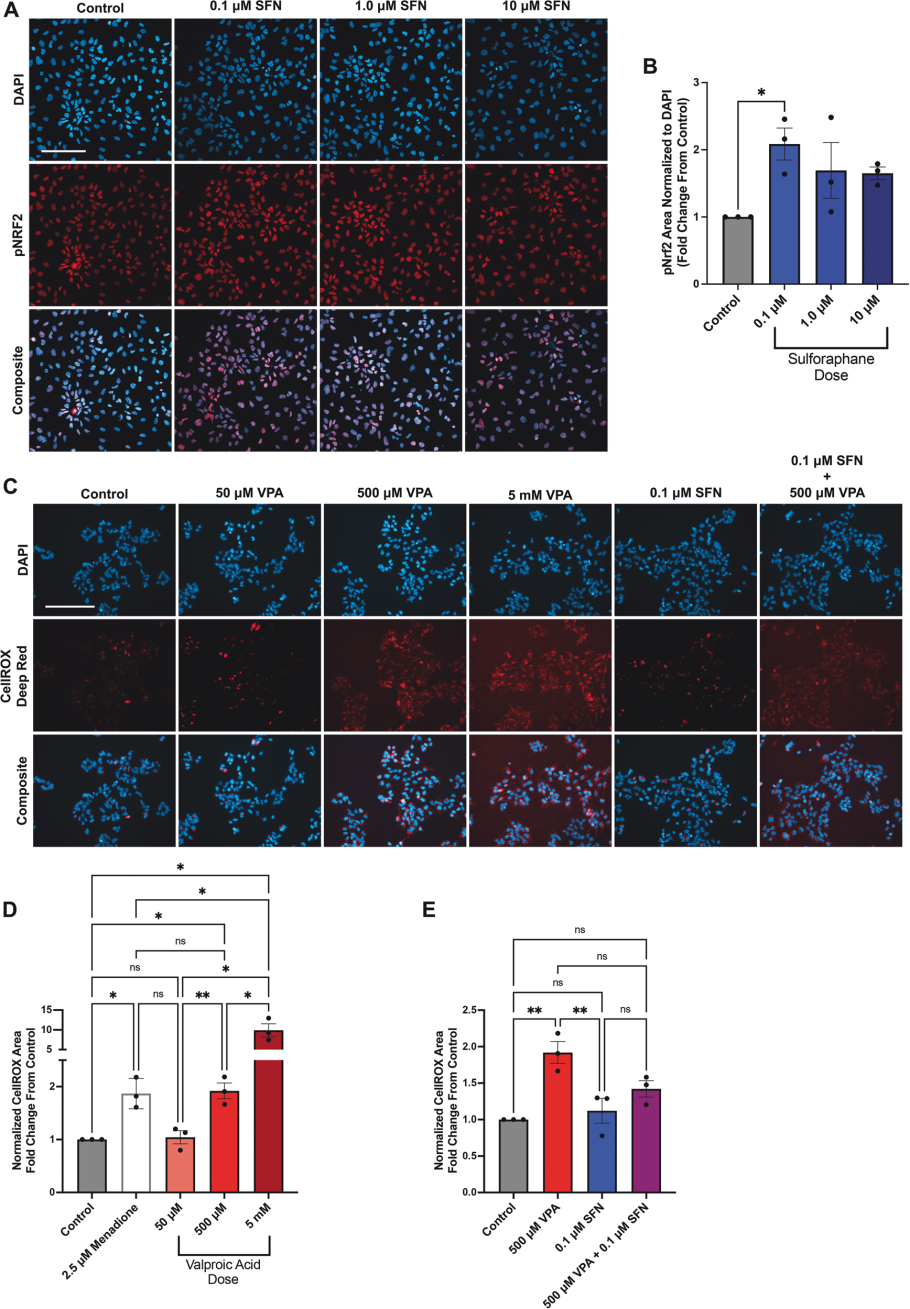
SFN attenuated VPA-induced ROS production in hNPCs. **A** Representative confocal images of phosphorylated NRF2 (pNRF2) (red) and DAPI counterstain (blue) in hNPCs with increasing doses of SFN. White scale bar represents 100 μm. **B** Quantified pNRF2 area normalized to DAPI represented as fold change from control (N = 3 experiments; analyzed using one-way ANOVA (p = 0.0210) with Dunnett’s multiple comparisons test). **C** Representative widefield images of CellROX™ Deep Red indicating cellular ROS in hNPCs treated with increasing concentrations of VPA, 0.1 μM SFN, and co-treated with 0.1 μM SFN and 500 μM VPA. White scale bar represents 200 μm. **D** Quantified CellROX area normalized to DAPI area expressed as fold change from control group for VPA dose response with 2.5 μM positive menadione control (N = 3 experiments; analyzed using one-way ANOVA (p < 0.0001) with Uncorrected Fisher’s LSD Test). **E** Normalized CellROX area fold change for 500 μM VPA alone, 0.1 μM SFN alone, and co-treatment with 500 μM VPA and 0.1 μM SFN (N = 3 experiments; analyzed using one-way ANOVA (p = 0.0039) with Tukey’s multiple comparisons test). Data represented as mean ± SEM, **p < 0.01 and *p < 0.05.

To select a SFN dose that increases NRF2 nuclear recruitment, we first screened increasing log-fold concentrations from 0.1–10 μM SFN in human neural progenitor cells (hNPCs), which model early neurodevelopment. We used a hNPC protocol first described by Brennand et al. to generate forebrain-patterned, proliferative hNPCs, that have the capacity to differentiate into either neurons or astrocytes, as we and others have previously shown [62–65]. While all SFN doses trended towards increased pNRF2 nuclear expression (Fig. 1A), only the lowest dose of 0.1 μM SFN significantly increased the expression of nuclear pNRF2, as measured by the total pNRF2 area normalized to the nuclear DAPI area (Fig. 1B). Interestingly, SFN did not increase the percentage of cells expressing pNRF2 (S. Fig. 1A) but rather increased the expression of pNRF2 within these cells as measured by the amount of nuclear area occupied by pNRF2 (S. Fig. 1B). These results suggest that hNPCs have similar capacity to engage NRF2 transcriptional pathways. Importantly, we did not observe changes in the total levels of NRF2 protein (S. Fig. 1C, D), demonstrating that increased pNRF2 is due to post-translational modification of the existing NRF2 pool. Notably, while high doses of SFN can exhibit cytotoxic effects [66, 67], low doses of SFN consistently demonstrate protective effects, especially in combination with cellular insults that increase oxidative stress [66, 68, 69]. Consistent with our findings, concentrations as low as 0.1 μM SFN protected rat striatal neurons from cell death from exposure to either H_2_O_2_ or paraquat [66]. Thus, we sought to test whether 0.1 μM SFN could protect against VPA-induced oxidative stress and subsequent neural circuit alterations associated with ASD.

Since a single dose of VPA is sufficient to produce autism-like behavior in rodent models [50], we sought to determine the acute VPA dose that would significantly upregulate cellular oxidative stress in our human neurodevelopmental model. Thus, we exposed human neural progenitor cells (hNPCs) to increasing doses of VPA from 50 μM to 5 mM, representing concentrations of VPA below, within, and above the therapeutic range used to treat epilepsy [70]. To identify an appropriate VPA dose, hNPCs were treated with VPA for 24 h followed by imaging of cellular oxidative stress with CellROX™ Deep Red, a commercially available fluorescence-based probe that produces a fluorescent signal in the presence of cellular ROS (Fig. 1C). Quantification of the fluorescent signal showed a dose-dependent increase in CellROX expression with increasing VPA concentrations (Fig. 1D). CellROX expression was calculated as the area of CellROX normalized to DAPI area, to account for differences in cell number. While 50 μM VPA was indistinguishable from control (50 μM VPA 1.043 ± 0.2144-fold change from control), we observed a noticeable increase in CellROX expression with 500 μM (1.919 ± 0.2587-fold change from control) that was further increased with 5 mM VPA (9.883 ± 2.827-fold change from control). Further, we found that 500 μM VPA and a standard menadione positive control for oxidative stress similarly (500 μM VPA 1.919 ± 0.2587 and 2.5 μM menadione 1.869 ± 0.2877-fold change from control) and significantly (500 μM VPA vs control p = 0.0254; menadione vs control p = 0.0347; 500 μM VPA vs Menadione p = 0.8756) increased cellular oxidative stress relative to the control (Fig. 1D and S. Fig. 1E). Thus, for our current studies, we selected 500 μM to model VPA-induced oxidative stress. Similar concentrations (500 μM–1 mM) have been used previously to model ASD in cell culture models including human forebrain organoids [59–61] and rodent neural progenitor cells [71].

**Fig. 2:**
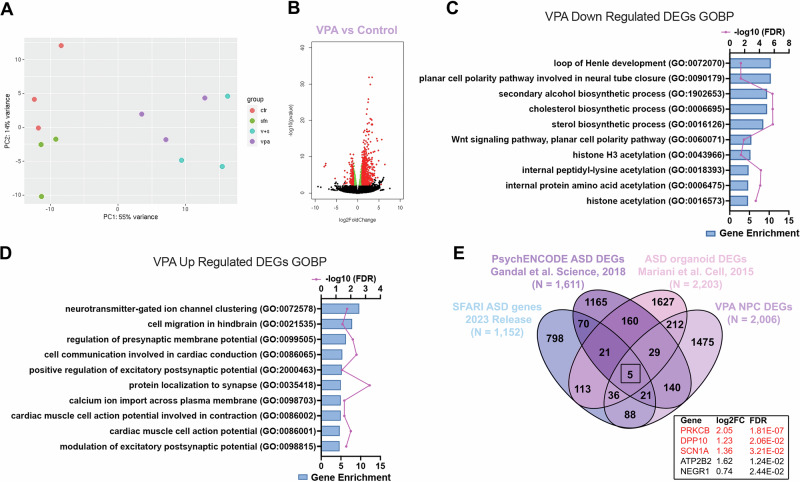
Acute VPA exposure led to ASD associated transcriptional changes in human neural progenitor cells. **A** Principal component analysis of hNPCs in control, 500 μM VPA, 0.1 μM SFN, and 500 μM VPA and 0.1 μM SFN co-treatment groups. **B** Volcano Plot of VPA vs Control showing statistically significant differentially expressed genes (DEGs) (padj > 0.05) above (red) and below (green) a threshold of log2fold change = 0.5 and unchanged genes (black). **C, D** Gene Ontology Biological Processes (GOBP) analysis of VPA-induced DEGs ordered by gene enrichment (ratio of the number of genes differentially expressed compared to total number of genes associated with that biological function) for down regulated **C** and up regulated **D** transcripts. **E** Overlap of VPA DEGs with ASD associated genes (SFARI), PsychENCODE ASD DEGs, and DEGs in ASD organoids derived from patient IPSCs.

We next tested whether 0.1 μM SFN decreased oxidative stress in the presence or absence of 500 μM VPA. In both cases, 0.1 μM SFN maintained cellular ROS close to control levels (SFN 1.121 ± 0.2981 and VPA + SFN 1.421 ± 0.1935-fold change from control), attenuating VPA-induced ROS production (VPA + SFN vs VPA p = 0.0918; VPA + SFN vs control p = 0.1661) (Fig. 1C, E). This robust effect warranted our continued use of 0.1 μM SFN in subsequent experiments to address whether SFN antioxidant buffering protects developing neural circuits. Notably, all CellROX expression measurements were normalized to cell count with the DAPI area, and we did not observe any changes in hNPC proliferation under these treatment conditions, as measured by expression of the proliferative marker Ki-67 (S. Fig. 2). Thus, our selected VPA dose alters cellular oxidative stress without confounding effects on cell survival. This is particularly important for modeling idiopathic autism spectrum disorders, which in most cases do not exhibit consistent gross anatomical changes, but rather altered neural connectivity at synapses [72].

### Neuroprotective signatures of sulforaphane

To elucidate neuroprotective pathways underlying this robust SFN-mediated antioxidant response, we performed unbiased bulk RNA sequencing of hNPCs treated with either vehicle control, 500 μM VPA, 0.1 μM SFN, or 500 μM VPA + 0.1 μM SFN in combination. Importantly, we selected hNPCs for our transcriptomic analysis rather than cortical spheroids, as they represent a homogenous cell population for the identification of genetic changes associated with neurodevelopment. Importantly, hNPCs also exhibit conservation of disease-associated gene expression changes with hIPSC-derived neurons [73], making them an ideal model to capture transcriptional signatures associated with early neurodevelopmental pathways. Following bulk RNA sequencing, principal component analysis (PCA) showed close clustering of individual replicates with their respective group (Fig. 2A), suggesting that the observed effects were due to treatment. Between groups, SFN treatment most closely clustered with control replicates, suggesting minimal impact of SFN alone on the cellular transcriptome. However, both VPA and VPA + SFN groups clustered distinctly from controls, but closely to each other (Fig. 2A). This close association could be due to the fact that VPA and SFN similarly function as HDAC inhibitors [74, 75]. Differential gene expression analysis between VPA-treated and control neural progenitor cells identified 2006 differentially expressed genes (DEGs) with a false discovery rate (FDR) < 0.05 (Fig. 2B). We first examined biological functions associated with up- and down-regulated mRNA transcripts for each treatment group using PANTHER GO Enrichment Analysis (S. Table 1) [76]. VPA decreased abundance of mRNA transcripts associated with biological processes involved in neural tube development and closure, Wnt signaling pathways, alcohol and sterol metabolism, and histone acetylation (Fig. 2C), but increased expression of genes related to synapse organization, cell migration and communication, as well as regulation of membrane potential (Fig. 2D). These transcriptome results are consistent with other cellular VPA models [59, 61] as well as the known teratogenic effects of VPA, such as neural tube closure defects. To further validate our model of chemically induced ASD, we used the analysis described previously by Meng et al. to compare their VPA-treated organoids to known ASD-associated genetic changes [59]. Comparison of DEGs from our VPA-treated hNPCs with known ASD genes from the Simon Foundation Autism Research Initiative (SFARI) (https://gene.sfari.org/), PsychENCODE ASD patient postmortem brains [77], and brain organoids derived from ASD patients [78], revealed extensive overlap and identified five overlapping genes across all four datasets, remarkably similar to the eight overlapping genes in the previously published organoid dataset with chronic VPA exposure, which also included *PRKCB, DPP10*, and *SCN1A* (Fig. 2E and S. Table 2). Thus, using hNPCs, we assessed transcriptomic changes in a homogenous cell population that uncovered significant alterations in neural-specific pathways that serve as the foundation for altered brain development.

**Fig. 3:**
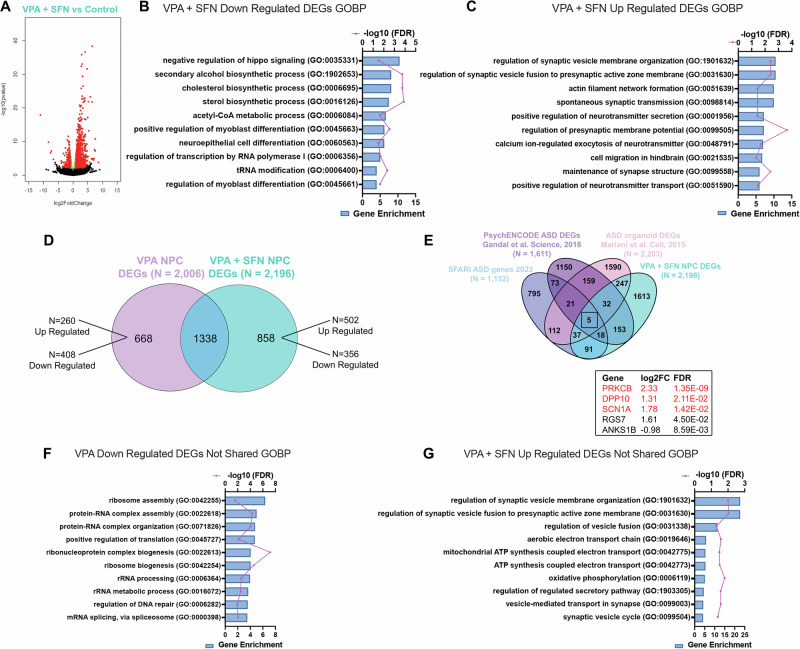
The addition of SFN increases transcription of genes associated with synaptic structure, organization, and mitochondrial metabolic processes. **A** Volcano Plot of hNPCs treated with VPA + SFN vs Control showing statistically significant DEGs above (red) and below (green) a threshold of log2fold change = 0.5 and unchanged genes (black). **B, C** GOBP analysis of DEGs in VPA + SFN treated hNPCs ordered by gene enrichment (ratio of the number of genes differentially expressed compared to total number of genes associated with that biological function) for down regulated **B** and up regulated **C** transcripts. **D** Comparison of the DEGs in VPA and VPA + SFN treated hNPCs showing 1338 shared DEGs between the data sets. **E** Overlap of VPA + SFN DEGs with ASD associated genes (SFARI), PsychENCODE ASD DEGs, and DEGs in ASD organoids derived from patient IPSCs. **F** GOBP analysis of the 408 DEGs down regulated in VPA treated hNPCs not shared with the VPA + SFN treatment. **G** GOBP analysis of the 502 DEGs up regulated in VPA + SFN treated hNPCs not shared with VPA elucidating potential mechanisms of SFN neuroprotection.

Having validated our model of chemically induced ASD, we sought to determine which gene expression alterations occurred in the presence of SFN, allowing it to significantly reduce the burden of cellular oxidative stress in VPA-treated hNPCs (Fig. 1). Overall, we uncovered 2196 DEGs when the VPA + SFN group was compared to the control (FDR < 0.05) (Fig. 3A). VPA + SFN decreased abundance of mRNA transcripts associated with hippo signaling, alcohol and sterol metabolism, cell differentiation, and RNA transcription and translation (Fig. 3B). While there was extensive convergence between the VPA and VPA + SFN treatment groups, there were also divergent biological pathways, with the addition of SFN notably increasing mRNA transcripts related to the maintenance of synapse structure and function (Fig. 3C). Comparison of VPA and VPA + SFN revealed a total of 1338 DEGs shared between VPA- and VPA + SFN-treated hNPCs (Fig. 3D). These expression profiles were also highlighted by comparison of VPA + SFN-treated hNPCs with known ASD datasets, as previously done for VPA treatment alone. VPA + SFN similarly altered expression of known ASD genes, *PRKCB, DPP10*, and *SCN1A* (Fig. 3E and S. Table 2). Notably, the *PRKCB* gene encodes protein kinase C Beta, which is involved in NRF2-mediated antioxidant response by direct phosphorylation of NRF2, inducing NRF2 nuclear translocation [79]. Isolation of DEGs specific to the VPA treatment resulted in 668 DEGs of which 260 were upregulated and 408 were downregulated (Fig. 3D). While GOBP analysis yielded no significant pathways associated with the upregulated genes, the significantly downregulated mRNA transcripts were associated with GOBPs for ribosome assembly, translation, and rRNA processing (Fig. 3F). Importantly, the addition of SFN restored many of these pathways back to control physiological levels (S. Table 1). To further examine the potential neuroprotective mechanisms of SFN, we focused our GO pathway analysis on the differentially expressed mRNA transcripts specific to VPA + SFN. This left us with 858 DEGs of which 502 were upregulated and 356 were downregulated with co-treatment of VPA and SFN compared to the control group (Fig. 3D). Intriguingly, SFN addition upregulated transcripts involved in synapse organization. Interestingly, mRNA transcripts associated with mitochondrial respiration were increased in hNPCs treated with VPA + SFN that were not significantly upregulated with VPA treatment alone (Fig. 3G). This finding is similar to other studies, which reported SFN promotes mitochondrial biogenesis and improves cellular metabolism [80–82].

Finally, we found the transcription of both direct and indirect NRF2-ARE mediated antioxidant enzymes were upregulated with co-treatment of VPA + SFN compared to VPA alone (S. Table 2). Notably, the expression of superoxide dismutase 3 *(SOD3)*, an enzyme responsible for the breakdown of superoxide radicals [83], was increased (2.19 log2fold change from control, p = 0.0003) with co-treatment compared to VPA alone (1.55 log2fold change from control, p = 0.008) highlighting the additive NRF2 antioxidant response of VPA-induced oxidative stress and SFN. Furthermore, we observed an increase in glutathione peroxidase *(GPX3)* mRNA transcription with co-treatment compared to VPA alone (1.35 log2fold change from VPA alone, p = 0.0091). This enzyme is highly expressed within the brain and protects cells from oxidative stress by catalyzing the reduction of hydrogen peroxide by the antioxidant glutathione [84]. While NRF2 binding at the ARE located in the promoter region for this gene has not been established, there is experimental evidence that it is transcriptionally regulated by peroxisome proliferator-activated receptor gamma *(PPARγ)* which is a NRF2 target gene [85] found to be significantly upregulated with co-treatment of VPA and SFN (2.66 log2fold change from control, p = 0.0049). Overall, these gene expression signatures highlight SFN’s potential to promote NRF2-mediated antioxidant pathways, cell survival, and synaptic preservation.

### Low dose sulforaphane robustly activates NRF2 in human cortical spheroids

The robust VPA-induced changes in neural-specific genes, particularly those related to synapses, led us to evaluate whether SFN can protect vulnerable developing neural circuits from VPA-induced synaptic alterations. To address the impact on synapse formation, we developed human cortical spheroids (hCSs) that resemble the developing dorsal forebrain at mid-fetal gestation [86]. HCSs provide us with the unique ability to model a sensitive period of brain development when synapses form and which is particularly susceptible to environmental disruption. We have previously used hCSs to research the impact of genetic and pharmacological manipulation on developing neural circuits [62, 87, 88].

**Fig. 4:**
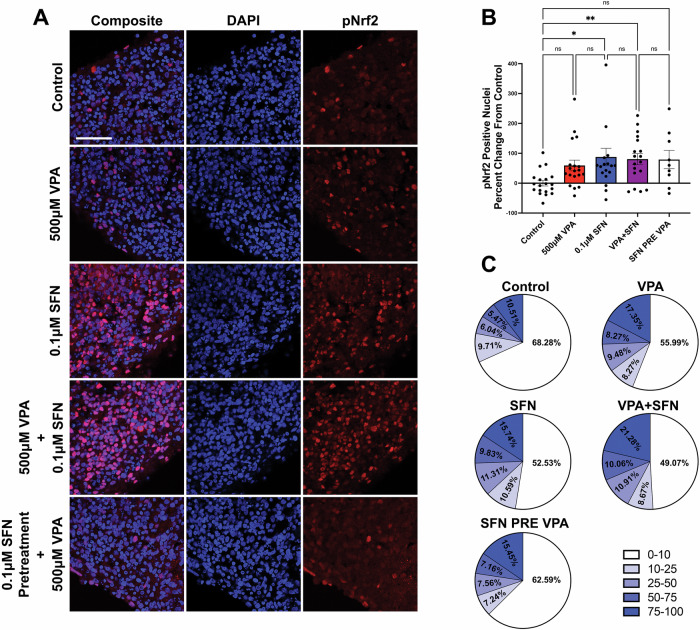
SFN treatment increased pNRF2 nuclear accumulation in hCSs. **A** Representative confocal microscopy images of DAPI stained nuclei (blue) and pNRF2 (red) in human cortical spheroids (HCS) treated with VPA, SFN, VPA and SFN or VPA pre-treated with SFN (scale bar represents 50 μm). **B** Quantification of pNRF2 positive nuclei in each treatment group compared to control (Data represented as Mean ± SEM; N = 18 for control, VPA, SFN, and VPA + SFN, N = 9 for SFN pre-treatment + VPA; data analyzed using Kruskal-Wallis test (p = 0.0026) with Dunn’s multiple comparisons test; **p < 0.01 and *p < 0.05). **C** Percent distribution of pNRF2 positive and negative nuclei for all cells. Positive nuclei correlate to greater than 10% overlap with DAPI divided into groups with 10–25, 25–50, 50–75, or 75–100% overlap.

We first validated NRF2 activation within this more complex and physiologically relevant model of brain development. After 90 days in culture when we observe extensive glutamatergic synapse formation [62, 87, 88], hCSs were exposed to either vehicle control, 500 μM VPA, 0.1 μM SFN, or 500 μM VPA and 0.1 μM SFN simultaneously in combination or with SFN pre-treatment followed by VPA. After 24 h of treatment, the hCSs were processed for immunostaining and imaged via confocal microscopy to measure nuclear pNRF2 accumulation (Fig. 4A). In our analyses, we quantified the total pNrf2 area relative to DAPI area (S. Fig. 3) and further quantified pNRF2 nuclear translocation in response to VPA and SFN treatments as percent area of the DAPI-stained nuclei (Fig. 4B). This allowed us to define a pNRF2 positive cell as one in which pNRF2 occupied ≥10% of the nuclear area, reducing false positives from background and overlapping nuclei. Cells with <10% pNRF2 nuclear area were considered negative. Our analyses revealed that VPA treatment led to a subtle, yet not statistically significant, increase in pNRF2-positive nuclei (control vs VPA, p = 0.1351) (Fig. 4B). Although the KEAP1-NRF2 interaction facilitates rapid NRF2 activation under conditions of oxidative stress, VPA has been demonstrated to dysregulate the NRF2 antioxidant response by downregulating NRF2 and target gene expression [56–58]. Therefore, this subtle increase may be indicative of aberrant alterations to NRF2 pathway functions in VPA-exposed cultures. Our analysis also revealed that exposure to SFN alone or in combination with VPA caused a statistically significant increase of ~80% from control (control vs SFN, p = 0.0139; control vs VPA + SFN, p = 0.0038). Interestingly, SFN pre-treatment followed by VPA exposure did not produce a statistically significant difference from either the control group or simultaneous administration (control vs SFN PRE VPA, p = 0.0906; VPA + SFN vs SFN PRE VPA, p > 0.9999). To further analyze the extent of NRF2 activation, we generated pie charts that subdivided the positive pNRF2 nuclear area into 10–25, 25–50, 50–75, and 75–100% of the total nuclear area. This revealed that VPA + SFN robustly increased active pNRF2 in the nucleus by predominantly increasing the fraction of nuclei with 75–100% pNRF2 accumulation, which doubled from control conditions (Fig. 4C). This data is consistent with our transcriptomic data, where we observed significantly increased expression of NRF2-related mRNA transcripts in the presence of SFN when compared to VPA alone (S. Table 2).

### Sulforaphane prevents VPA-induced alterations of glutamatergic synapses

**Fig. 5:**
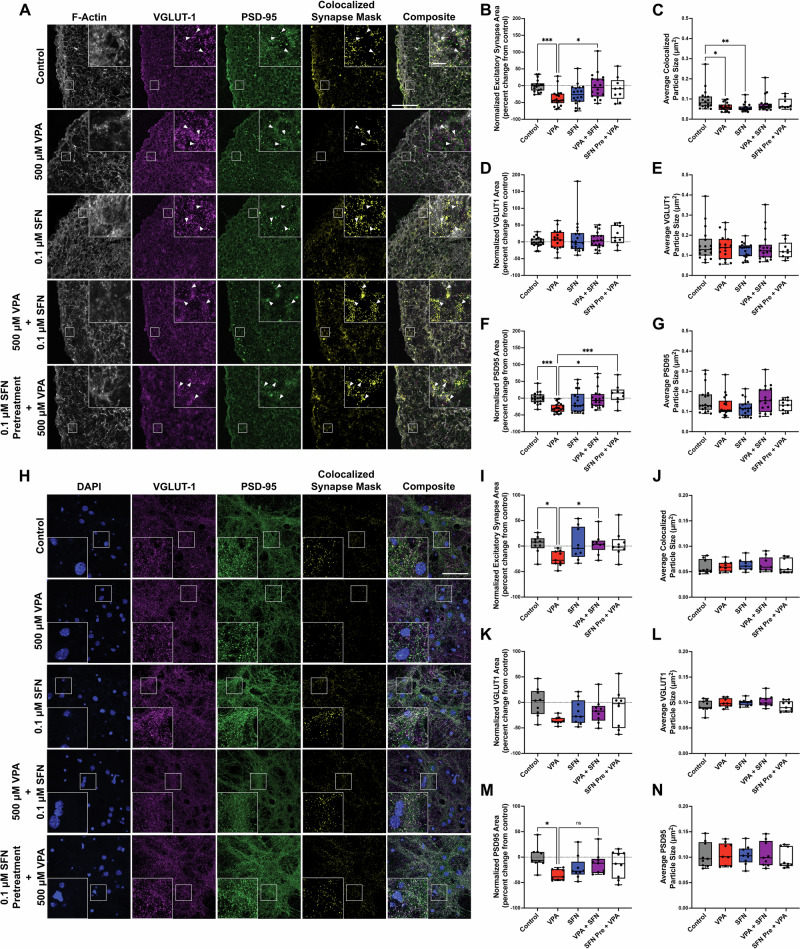
SFN mediated VPA-induced glutamatergic synaptic alterations. **A** Representative confocal images of human cortical spheroids (hCSs) labeled with excitatory presynaptic marker VGLUT-1 (magenta) and postsynaptic marker PSD-95 (green) with fluorescently labeled actin (white) after 24-h treatment of 500 μM VPA, 0.1 μM SFN, 500 μM VPA and 0.1 μM SFN, or 24-h SFN pre-treatment followed by 24-h treatment with 500 μM VPA. Arrowheads indicate colocalization of VGLUT-1 and PSD-95 also shown as colocalized synapse mask (yellow). White scale bar in the control composite represents 50 μm in original image and 5 μm for inset of the enlarged ROI. All images are set to the same scale. **B-G** for each image we quantified the **B** normalized VGLUT-1/PSD-95 colocalized puncta (excitatory synapse) as area percent change from control, **C** VGLUT-1/PSD-95 colocalized puncta particle size, **D** normalized VGLUT-1 puncta area as percent change from control, **E** VGLUT-1 puncta particle size, **F** normalized PSD-95 puncta area as percent change from control, and **G** PSD-95 puncta particle size. **H** Representative confocal images of mouse cortical neurons (mCNs) labeled with excitatory presynaptic marker VGLUT-1 (magenta) and postsynaptic marker PSD-95 (green) with DAPI (blue) after 24-h treatment of 500 μM VPA, 0.1 μM SFN, 500 μM VPA and 0.1 μM SFN, or 24-h SFN pre-treatment followed by 24-h treatment with 500 μM VPA. White scale bar in the control composite represents 50 μm in original image and the inset is a 2.5× enlargement of the ROI. **I-N** for each image we quantified the **I** excitatory synapse area per nuclei as percent change from control, **J** VGLUT-1/PSD-95 colocalized puncta particle size, **K** normalized VGLUT-1 puncta area as percent change from control, **L** VGLUT-1 puncta particle size, **M** normalized PSD-95 puncta area as percent change from control, and **N** PSD-95 puncta particle size. Data were analyzed with Kruskal-Wallis test, **B** p = 0.0005; **C** p = 0.0026; **D** p = 0.6369; **E** p = 0.9571 **F** p < 0.0001**; G** p = 0.1739), **I** p = 0.0238; **J** p = 0.6889; **K** p = 0.0931; **L** p = 0.1473 **M** p = 0.0259**; N** p = 0.8761), followed by Dunn’s multiple comparisons test when p < 0.05 to determine statistical significance. In hCS data, N  =  18 micrographs, 3 each from 6 independent experiments analyzed per treatment group except for the 0.1 μM SFN pre-treatment + 500 μM VPA, which had N = 9 micrographs, 3 each from 3 independent experiments. In mCN data, N  =  9 micrographs, 3 each from 3 independent experiments analyzed per treatment group. ***p < 0.001, **p < 0.01, and *p < 0.05.

In addition to upregulating NRF2-related pathways, our RNA-sequencing results revealed that the addition of SFN to VPA-treated cells also significantly upregulated synaptic mRNA transcript levels (Fig. 3 and S. Table 1). We also validated 3 downregulated and 3 upregulated genes from our hNPC RNA-seq in VPA-treated hCSs (S. Fig. 4). To determine whether SFN made synapses resilient to the detrimental effects of VPA, we analyzed the formation of glutamatergic synapses in hCSs exposed to VPA and SFN either alone or in combination. Using confocal microscopy, we imaged and quantified excitatory pre- and post-synaptic markers vesicular glutamate transporter 1 (VGLUT-1) and postsynaptic density protein 95 (PSD-95) respectively in our hCSs. The area in which the pre- and post-synaptic markers overlapped was designated as a synapse (Fig. 5A and S. Fig. 5). The total glutamatergic synapse area (Fig. 5B) and the size of individual synapses (Fig. 5C) were significantly reduced in hCSs treated with VPA compared to control (control vs VPA global area, p = 0.0008; control vs VPA puncta, p = 0.0335), similar to recent observations of reduced synapse formation during early brain development of non-human primates exposed to VPA [89].

While SFN alone did not significantly alter total synapse area (Fig. 5B, control vs SFN, p = 0.0514), we observed a reduction in the average size of colocalized excitatory synapse puncta compared to control (Fig. 5C, control vs SFN, p = 0.0021). Notably, SFN administered simultaneously prevented VPA-induced synaptic deficits in synapse area (Fig. 5B, VPA vs VPA + SFN simultaneous treatment, p = 0.0151; control vs SFN + VPA simultaneous treatment, p > 0.9999). Similarly, when SFN was administered as a pre-treatment, there were no significant changes in synapse area compared to control (Fig. 5B, control vs SFN pre-treat + VPA, p > 0.9999). Thus, while SFN pre-administration did not persistently elevate NRF2, the synaptic outcomes were similar to simultaneous SFN administration with VPA.

To determine whether VPA-induced synaptic changes were driven by pre- or post-synaptic alterations, we also examined VGLUT-1 and PSD-95 individually. While we measured no significant changes in pre-synaptic (VGLUT-1) area or particle size between any treatment groups (Fig. 5D, E), VPA reduced post-synaptic (PSD-95) area compared to control (p = 0.0108), which was rescued by SFN either administered simultaneously or as a pre-treatment (VPA vs VPA + SFN simultaneous treatment, p = 0.0106; VPA vs SFN pre-treat + VPA, p = 0.0007) (Fig. 5F). No significant changes in PSD-95 particle sizes were seen in any treatment groups (Fig. 5G), implicating reduced formation of post-synaptic specializations, rather than a reduction in individual post-synapse size. This finding supports early reductions of dendritic spine density in non-human primates with prenatal VPA exposure [89] as well as reduced PSD-95 observed in early postnatal development of VPA-exposed mice [90].

To determine whether these synaptic effects were conserved across species, we treated embryonic (e)-18.5 mouse primary cortical neurons with the same treatment groups and fixed at day in vitro (DIV)-16, when synapses have formed, but have not yet fully matured [91] (Fig. 5H). As with human cortical spheroids, VPA significantly reduced excitatory synapse formation (VPA vs control p = 0.0409), which was prevented by the addition of SFN (VPA vs VPA + SFN p = 0.0484; Fig. 5H, I), and this effect was also driven by a significant reduction in post-synaptic PSD-95 area (VPA vs Control p = 0.0140; Fig. 5M). However, no effects on the size of individual synapses consisting of co-localized puncta of VGLUT-1 and PSD-95 or individual VGLUT-1 pre-synaptic or PSD-95 post-synaptic puncta were observed in any of the treatment groups (Fig. 5J, L, and N). These results demonstrate that SFN confers synaptic resilience in cortical neurons, regardless of species.

### Sulforaphane rescues VPA-induced changes in synaptic activity in developing neural networks

**Fig. 6:**
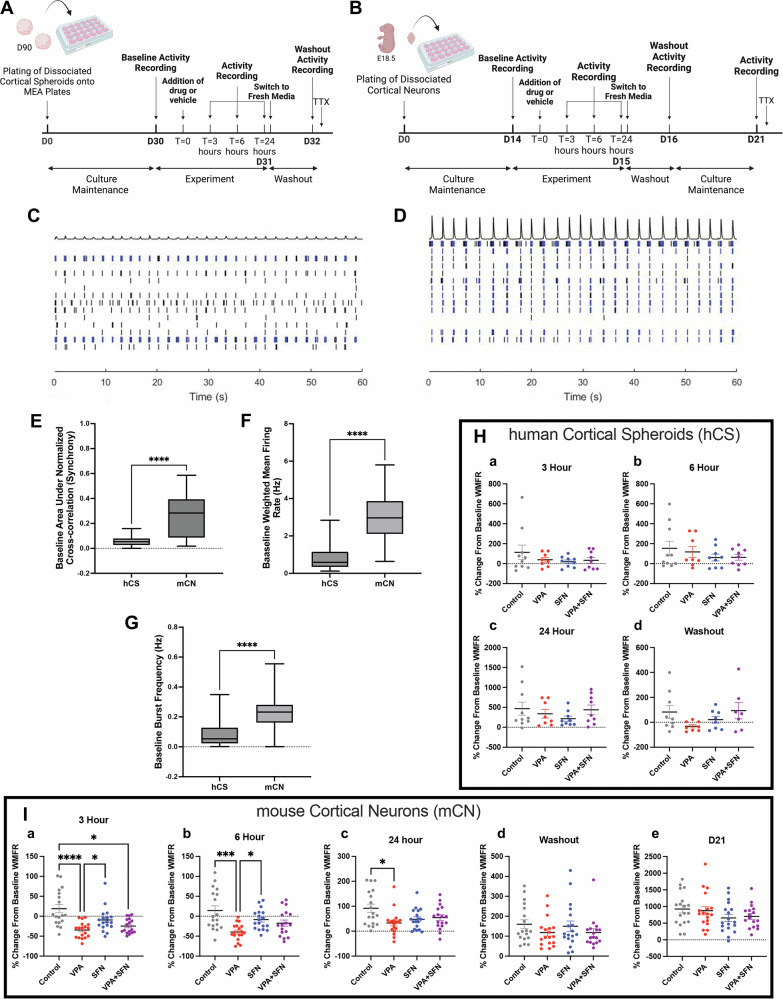
Sulforaphane rescues VPA-induced changes in synaptic activity in developing neural networks. **A** Detailed schematic illustrating human cortical spheroid (hCS) experimental protocol for microelectrode array (MEA). **B** Detailed schematic outlining primary mouse cortical (mCN) experimental protocol for MEA. **C, D** Representative raster plots of hCS **C** and mCN **D** baseline activity measurements taken prior to drug treatment, black bars represent a single spike and blue bars represent bursts in which more than 5 action potentials occur with spikes no more than 100 ms apart. **E–G** Quantification of baseline synchrony metrics **E** computed in the neural metric tool as the normalized area under the cross-correlation, weighted mean firing rate (WMFR) **F**, and bursting frequency **G** for each culture. Data were analyzed using Mann-Whitney test (**E-G** p < 0.0001) (hCS N = 56; mCN N = 71 MEA wells). **H** hCS WMFR quantified for each treatment at 3 h **H.a**, 6 h **H.b**, 24 h **H.c**, and 24-h washout **H.d** normalized to baseline WMFR. Data were analyzed using Kruskal-Wallis test (all p > 0.05, N = 8–10 MEA wells per treatment). **I** mCN WMFR quantified for each treatment at 3 h **I.a**, 6 h **I.b**, 24 h **I.c**, 24-h washout **I.d**, and 21 (days in vitro) DIV **I.e** normalized to baseline WMRF. Data were analyzed using Kruskal-Wallis test with Dunn’s multiple comparison when p < 0.05, N = 17 or 18 MEA wells per treatment. Data represented as mean ± SEM, ****p < 0.0001, ***p < 0.001, **p < 0.01, and *p < 0.05.

Our hCS model of prenatal VPA exposure elicited reductions in glutamatergic synapse formation that were attenuated by co-administration of SFN (Fig. 5). With this work, we have demonstrated SFN’s ability to protect developing synapses from VPA-induced alterations. To address whether SFN’s ability to prevent changes in synaptic structure also confers protection of neuronal function, we employed microelectrode array (MEA) analysis to assess changes in spontaneous action potential formation. After 90 days in culture, hCSs were dissociated onto MEA plates and allowed to reform connections and establish consistent activity over 30 days (Fig. 6A). We have previously used this dissociation model as it facilitates better contact between neurons and the electrodes [62, 87, 88]. On day 30, we recorded the baseline activity of the neural networks prior to the addition of either control media, 500 μM VPA, 0.1 μM SFN, or VPA + SFN. We then recorded the neural activity after 3, 6 and 24 h as well as 24 h after washout (see Fig. 6A for a detailed workflow). We similarly assessed neural circuit activity in primary mouse cortical neurons (see Fig. 6B for a detailed workflow). To obtain our primary cultures, cortical brain regions were carefully dissected from e18.5 mice, dissociated, and plated onto MEA plates. Due to their increased developmental maturity, the primary mouse cortical neurons (mCNs) established consistent activity after only 14 days in culture, which corresponds to our analysis of synapse structure (Fig. 5). At this time, we performed the same experimental recordings described for the dissociated hCSs (Fig. 6B).

To analyze changes in neural activity, we normalized the weighted mean firing rate (WMFR) of each well back to the baseline WMFR prior to treatment. The raster plots of the well display the activity recorded from each electrode in a horizontal line. In the raster plots (Fig. 6C, D), spikes corresponding to spontaneous action potentials are shown as black lines and electrode bursts of multiple spikes in succession are shown in blue. Comparison of baseline neural activity between dissociated hCSs and mCNs shows that mCNs have significantly higher synchrony across electrodes (Fig. 6E) (synchrony mCN vs hCSs, p < 0.0001). Additionally, mCNs exhibit increased WMFR (Fig. 6F) and burst frequency (Fig. 6G) when compared to hCSs (WMFR mCN vs hCSs, p < 0.0001; burst frequency mCN vs hCSs, p < 0.0001). All these parameters indicate increased maturation of neural circuit activity in mCNs when compared to hCSs. Consistent with this maturation, control mCNs exhibited less change in activity throughout the 24-h recording schedule, whereas control hCSs were more variable in activity. Due to the lower maturation of hCS neural circuits, we observed no statistically significant differences in WMFR across treatment conditions (Fig. 6H). Intriguingly, 24 h after washout, the VPA treatment trended downwards, although not significantly (Fig. 6H.d). These data warrant further investigation into whether VPA-induced synaptic alterations in immature developing circuits will have subsequent activity alteration as neural circuits mature. However, loss of hCS neuronal viability with continued culture on MEA plates prevented us from addressing these long-term effects in the present study.

However, in the more mature mCN circuits, VPA significantly reduced neural activity throughout the 24-h duration of the recording. After three hours of exposure to VPA, we observed a 34.61 ± 19.66% decrease in WMFR in mCNs compared to the baseline recording which was significantly lower than both control and SFN treated mCNs which exhibited an 18.96 ± 44.86% (control vs VPA, p < 0.0001) and a −9.47 ± 30.15% (SFN vs VPA, p = 0.0420) change from baseline respectively (Fig. 6I.a). At this time point, SFN alone had no significant effect on WMFR, while co-administration with VPA led to a −24.67 ± 18.49% change from baseline which was still significantly lower than the control (control vs VPA + SFN, p = 0.0116). The significant difference in WMFR between control and VPA treated mCNs was maintained for the duration of exposure (control vs VPA at 6 h, p = 0.0019; control vs VPA at 24 h, p = 0.055) (Fig. 6I.b-c). However after 6 h of treatment, SFN co-administration restored the WMFR (Fig. 6I.b), which continued for the remainder of the 24-h recording (Fig. 6I.c) Additionally, there were no statistically significant changes between any of the treatment groups after either the 24-h washout period or one week (D21) after the initial exposure, indicating that acute VPA exposure did not permanently reduce spontaneous action potential formation. Taken together, these results reveal a critical window of neural circuit development that is sensitive to chemical disruption by VPA and that SFN can provide significant neuroprotection.

## Discussion

ROS homeostasis is crucial in the maintenance of cell-cell signaling, physiological function, and prevention of cellular damage [7–9, 92]. This is especially relevant in high oxygen consuming organs such as the brain, where aberrant ROS production contributes to an increase in cellular dysfunction and ultimately increases the likelihood of neurodevelopmental disorders and cognitive decline. Cellular antioxidant pathways such as the NRF2-ARE pathway counteract excessive ROS levels by increasing antioxidant enzymes and cytoprotective proteins [22–25]. In recent years, there has been growing interest in the ability of naturally derived phytochemicals to combat aberrant oxidative stress through the activation of the NRF2-ARE pathway [26–28, 33–35]. In this study, we investigated whether the phytochemical SFN, a potent activator of the NRF2-ARE pathway, could protect developing neural networks from VPA-induced oxidative stress. Ultimately, our results demonstrated SFN’s ability to promote synaptic resiliency in the presence of VPA and identified key transcriptomic changes underlying this effect.

Importantly, like VPA, numerous other chemicals are known to promote ASD-associated gene signatures and upregulate oxidative stress [93]. These include common fungicides that upregulate superoxide production by inhibiting either mitochondrial complexes I or III [93]. These fungicides reduce expression of ion channels and synaptic genes [93]. Notably, in our RNA-sequencing dataset, SFN increased transcript levels of mitochondrial complex I as well as genes related to synapse formation (Fig. 3). Thus, SFN has the potential to protect developing synapses from a variety of environmental toxicants.

Our results also validate VPA’s ability to model chemically induced ASD in human neurodevelopmental models. Consistent with VPA-induced autism rodent models [94, 95], VPA increased cellular ROS in hNPCs (Fig. 1). Our experiments in these homogenous cell populations allowed us to analyze the transcriptome without confounding variability arising from complex multicellular models. However, RT-PCR of DEGs in HCSs show similar changes (S. Fig. 4). Importantly, the VPA-induced transcriptomic changes in our hNPC model of early neurodevelopment were similar to those observed by other research groups who repeatedly exposed human brain organoids to VPA [59–61]. These gene expression profiles were also shared with published DEGs in human ASD transcriptomes [77, 78] (Fig. 2E). Transcriptional similarities between VPA and VPA + SFN, particularly in ASD-associated genes (Figs. 2E, 3E), suggest that SFN protects synapses primarily through resolution of oxidative stress. These transcriptional similarities are consistent with VPA’s known role as a potent HDAC inhibitor, which induces aberrant gene expression (Fig. 2). Furthermore, VPA significantly decreased expression of genes in GO pathways related to ribosomal assembly/translation, many of which were restored to control expression levels by the addition of SFN (S. Table 1). This reduction in RNA translation/processing pathways is consistent with an integrated stress response, in which cells divert resources away from protein translation and cell growth to resolve oxidative stress from external insults [96]. SFN’s recovery of many (though not all) of these pathways to control levels further supports its potent antioxidant effects. In addition to restoring physiological processes, SFN increased expression of genes related to synapse structure and function, suggesting that SFN could preserve synaptic integrity in the presence of VPA.

While prenatal VPA exposure has long been associated with postnatal neural hyperexcitability and elevated glutamatergic signaling [97, 98], the effects of VPA on developing neural networks of the fetal and early post-natal periods have been understudied. These early developmental periods correspond to critical periods of synapse formation. In the developing rodent cortex, synapse formation peaks within the first month [99, 100]. Recent studies in rodents and primates have demonstrated that VPA initially decreases glutamatergic synapse formation and function in these developing neural networks [89, 90, 101]. Our findings are the first to demonstrate that acute VPA exposure similarly reduces glutamatergic synapse formation in a human forebrain model (Fig. 5A–G). Acute VPA exposure also reduced glutamatergic synapse formation in mouse embryonic cortical neurons (Fig. 5H–N). Thus, while most studies focused on postnatal periods following VPA exposure, our approach allows us to model understudied events of early neurodevelopment.

Using this model of chemically induced ASD, we identified transcriptomic signatures of SFN-mediated neuroprotection that conferred resistance to VPA-induced alterations in synapse structure and the emergence of neural network activity. Importantly, these beneficial effects in our neurodevelopmental models were seen using a low dose of SFN (0.1 μM, Figs. 1C, F and 3–6), which is important given that higher SFN doses have been shown to reduce cell viability in neuroblastoma cell lines at doses as low as 1.0 μM, with complete cell death at doses ≥15 μM [102]. This biphasic effect of SFN, known as hormesis, is well-documented in research initially stemming from the anti-cancer effects of SFN in reducing cell proliferation, migration and metastasis while increasing cell cycle arrest and apoptosis at high doses, versus enhancing cell viability and proliferation at lower doses [103, 104]. Notably, our low SFN dose minimally impacted cell physiology, and only revealed its beneficial properties when combined with VPA, suggesting safety and efficacy of SFN at low doses to protect developing neural networks. Importantly, more research is needed to assess the long-term safety and efficacy profile of SFN under chronic treatment conditions.

Neural network formation and the development of neural activity is a complex process that begins in humans during prenatal brain development and extends into juvenile periods [105]. Spontaneous activity increases during this time and becomes synchronized across connected cells. While synapse formation begins during mid-fetal gestation, neural activity is often immature and relies less on synaptic connections [106]. These synaptic connections will be the predominant sites of action potential formation after birth. However, in the prenatal and early postnatal brain, gap junctions play a larger role that will diminish with further brain development [106]. Consistent with this developmental trajectory, our experiments showed immature activity in hCSs. While we observed VPA-induced synaptic reductions in hCSs (Fig. 5), we found that VPA had no effect on spontaneous action potential formation in this model (Fig. 6H). Importantly, SFN’s preservation of synaptic structure in the presence of VPA at this early developmental time could prevent the emergence of subsequent activity alterations in more developed circuits, which is supported by SFN-mediated recovery of neural activity in more mature networks of mouse cortical neurons. Importantly, none of our treatments lead to lasting changes in neural activity, suggesting a chronic model is needed in future studies to assess persistent neural circuit alterations.

Ultimately, this research advances our understanding of the potential neuroprotective effects SFN can have against environmental risk factors during the sensitive period of brain development. We demonstrated SFN’s ability to confer resilience to nascent synaptic connections in the presence of VPA, preventing reductions in glutamatergic synapse formation. Further, we found SFN to be protective against neural circuit activity disruption by VPA. Together, these findings motivate future exploration of SFN’s neuroprotective mechanisms against other environmental and chemical insults.

## Materials and methods

### Cell lines and maintenance

The EGFP-tagged Paxillin (PXN) WTC induced pluripotent stem cell (iPSC) (Cell Line ID: AICS‑0005) and TagBFP-tagged d-Cas9-KRAB WTC iPSC (Cell Line ID: AICS‑0090‑391) lines were developed at the Allen Institute for (Cell Science Seattle, WA, USA) (allencell.org/cell-catalog) and available through Coriell (Camden, NJ, USA) [107]. iPSCs were maintained on 6 well plates coated with Geltrex hESC Matrigel (Fisher Scientific, Hampton, NH, USA, Cat# A141302) in mTeSR^TM^ Plus media (Stemcell Technologies, Vancouver, Canada, Cat# 100-0276) at 37 °C and 5% O_2_. Media were supplemented with 10 μM ROCK inhibitor Y-27632 (Selleck Chemicals, Houston, TX, USA/VWR, Radnor, PA, USA, Cat# 101763-964) during the first 24 h after thawing or passaging cells. All cultures underwent regular mycoplasma testing with MycoAlert PLUS detection kit (Lonza, Basel, Switzerland, Cat# LT07-710).

### Animals

All procedures using animals and animal material adhered to guidelines outlined in the National Research Council Guide for the Care and Use of Laboratory Animals and were approved by the Animal Care and Use Committee at East Carolina University (AUP A3469-01) for the laboratory of Dr. Karen Litwa. CD-1 timed pregnant dams were purchased from Charles River Laboratories (Wilmington, MA, USA).

### Ethics statement

All methods were performed in accordance with the relevant guidelines and regulations.

### Neural progenitor cell culture

Human neural progenitor cells (hNPCs) were generated from the TagBFP-tagged d-Cas9-KRAB WTC iPSC line following an embryoid body protocol from Brennand et al. [64], as described in our previous publication [108]. hNPCs were maintained at 37 °C with 5% CO_2_ on Matrigel-coated (Corning, Corning, NY, USA/Fisher Scientific, CB40234A) tissue culture plates with hNPC media consisting of Dulbecco’s Modified Eagle Medium/Nutrient Mixture F-12 (DMEM/F-12) + GlutaMAX (Fisher Scientific, Cat# 10-565-042) supplemented with N-2 (Gibco/Fisher Scientific, Cat# 17-502-048), B-27 without vitamin A (Gibco/Fisher Scientific, Cat# 12-587-010), 1 μg/mL laminin (Corning/VWR, Cat# RL610-1521215), and 20 ng/mL basic fibroblast growth factor (bFGF) (Shenandoah Biotechnologies, Warminster, PA, USA/VWR, Cat# 10821-972). To passage the cells, 500 μL/well of StemPro^TM^ Accutase^TM^ Cell Dissociation Reagent (Gibco/Fisher Scientific, Cat# A1110501) was used to gently remove the cells from plate. hNPCs were used in experiments between passages 6–15.

For dose response experiments, hNPCs were seeded at a density of 50,000 cells per well onto Matrigel-coated 12-well plates with #1.5 acid-washed glass coverslips (Thermo Fisher, cat# 22-050-244) (acid washed in 6 M hydrochloric acid, dried, and UV-light sterilized) and allowed to adhere overnight at 37 °C with 5% CO_2_. The next day, media were replaced with the respective treatment media and the cells were returned to the incubator for 24 h.

For RNA-sequencing, hNPCs were seeded onto Matrigel-coated 10 cm plates at the same density used in previous experiments with cell number adjusted for surface area. As done previously, cells were allowed to adhere overnight at 37 °C with 5% CO_2_ and the next day, the media were replaced with the respective treatment media and the cells were returned to the incubator for 24 h.

### Live cell CellROX ^TM^ assay

CellROX^TM^ Deep Red Reagent (Invitrogen/Fisher Scientific, Cat# C10422) was used according to manufacturer’s protocol at a final concentration of 5 μM and NucBlue^TM^ Live Cell Stain Ready Probes^TM^ reagent (Invitrogen/Fisher Scientific, Cat# R10477) was used at 1 drop/mL. In the final 30 min of our 24-h dose response experiment, the CellROX^TM^ and NucBlue^TM^ reagents were added, and the cells were returned to the incubator at 37 °C with 5% CO_2_. The cells were then washed three times with 1× phosphate-buffered saline (PBS) (VWR, Cat# VWRL0119-0500) and fresh hNPC media added before imaging with the EVOS^TM^ FL Auto 2 Imaging System (Invitrogen/Fisher Scientific, Cat# AMAFD2000) at 5% CO2 with >85% humidity. To image the CellROX^TM^ we used the CY5 Fluorescent Light Cube (Invitrogen/Fisher Scientific, Cat# AMEP4656) and DAPI Fluorescent Light Cube (Invitrogen/Fisher Scientific, Cat# AMEP4650). Triplicate images were taken of each well and analyzed in the open-source ImageJ platform Fiji [109]. CellROX^TM^ channel was held to constant threshold to collect area of signal above background. The data was then normalized to DAPI area to account for differing numbers of cells within respective imaging areas. Within each experimental replicate, the treated conditions were normalized to control for comparison between experiments. N = 3 experimental replicates.

### Immunostaining of hNPCs and Imaging

For immunostaining of treated hNPCs either on coverslips or in 24-well tissue culture plates, after 24 h of exposure, cells were washed three times with 1× phosphate-buffered saline (PBS) (VWR, Cat# VWRL0119-0500) and fixed with 4% paraformaldehyde (PFA) (Fisher Scientific, Cat# PI28908) and 4% sucrose (Fisher Scientific, Cat# A1558336) in 1× PBS for 20 min. The cells were rinsed three times with tris-buffered saline before blocking with 5% normal goat serum (NGS) (Sigma-Aldrich, St. Louis, MO, USA, Cat# S26) in 1× PBS for 30 min. Plates were then incubated overnight at 4 °C with either primary phospho-(p)NRF2 antibodies (Abcam, Cambridge, UK, phospho-Ser40, Rabbit monoclonal antibody, Cat# ab76026, RRID:AB_1524049, dilution 1:500) Nrf2 antibodies (Abcam, Rabbit monoclonal antibody, Cat# ab62352, dilution 1:200), or Ki67 antibodies (Abcam, Rabbit polyclonal antibody, Cat# ab15580, dilution 1:400) in 2% NGS in 1× PBS. The following day they were washed three times with 1× PBS followed by incubation at room temperature with secondary antibodies (Invitrogen/Fisher Scientific, Anti-Rabbit Alexa Fluor 647, Goat Polyclonal, Cat# A-21245, RRID:AB_2535813, dilution: 1:500) in 2% NGS in 1× PBS for one hour in the dark. The plates were washed twice with 1× PBS before counterstaining with DAPI diluted 1:1000 in 1× PBS for five minutes followed by 2 washes in 1× PBS. Coverslips were mounted with Fluoro-gel Mounting Medium with Tris Buffer (Electron Microscopy Sciences, Cat# 1798510) while 24-well plates received 500 μL fresh 1× PBS prior to imaging.

For pNRF2 and NRF2 experiments, images were acquired on the ZEISS LSM 900 confocal microscope with the 20×/0.8 M27 Plan Apochromat objective using the 639 and 405 channels. Z-stacked images were acquired from three areas around each coverslip with 3 z-planes taken over a total depth of 1.08 μm. The open-source ImageJ platform Fiji [109] was used for quantification and analysis of these images beginning with the generation of maximum intensity projections of each z-stack. Each image was converted to an 8-bit tiff file and all samples within the same experiment were held to the same DAPI threshold restriction. The watershed function on Fiji was used to isolate merged individual nuclei and particle analysis was used to count and create outlines of each nucleus and add each area to the ROI manager. These nuclear ROIs were used to measure pNRF2 signal within the nucleus and we collected the signal intensity as well as the percent area for our analysis of pNrf2 response. For our population analysis, we defined pNRF2 positive nuclei as an area fraction >10% to distinguish between baseline signal and biologically relevant nuclear translocation in hNPCs which have elevated baseline pNrf2. For hNPCs stained with NRF2, signal was held to constant threshold across conditions and total area was normalized to DAPI area. N = 3 experimental replicates.

Ki67 and DAPI stained hNPCs were imaged on the EVOS^TM^ FL Auto 2 Imaging System previously used for live cell imaging. Similarly, we used the CY5 Fluorescent Light Cube and DAPI Fluorescent Light Cube to capture triplicate images of each well and analyzed in the open-source ImageJ platform Fiji [109]. The Ki67 channel was held to a consistent threshold within each replicate and the Ki67 area fraction for each nucleus was recorded using the ROI manager as done with the pNRF2. N = 3 experimental replicates

### RNA extraction and gene expression

After 24-h exposure to treatments, hNPCs were lysed and RNA extracted in TRIzol Reagent (Fisher Scientific, Cat# 15-596-026) with cell scraping followed by RNA purification using Direct-zol RNA Miniprep Kit (Zymo Research, Irvine, CA, US Kit# R2052/VWR Cat# 76211-340) according to manufacturer’s instructions. RNA quality was examined by the 4200 TapeStation (Agilent Technologies, Santa Clara, CA, USA) and RNA concentration was determined using the Qubit Fluorometric Quantitation (Thermo Fisher Scientific). Stranded cDNA libraries were prepared using the TruSeq Stranded Total RNA prep kit (Illumina, San Diego, CA, USA) in accordance with the manufacturer’s protocol using the poly-adenylated RNA isolation. Sequencing of paired end reads (100 bp × 2) was performed on the NextSeq 2000 system (Illumina, San Diego, CA, USA).

For differential gene expression, sequence reads of each sample were pseudo-aligned to the human hg38 reference transcriptome and transcript abundance was quantified by using Kallisto (v.0.46.1). Differential gene expression analyses were achieved by using tximport (v1.20.0) and DESeq2 (v1.26.0) packages in R Studio (Build 351 with R v4.1.1). Gene ontology analysis was performed using the online GO Enrichment Analysis tool powered by the Panther Classification System [110, 111].

### Generation of three-dimensional cortical spheroids

We generated human cortical spheroids (hCSs) following methods developed and described by Pasca et al. [86]. Briefly, on day 0 confluent IPSCs were enzymatically lifted from culture dishes using Pluri-STEM Dispase-II (Millipore-Sigma, Burlington, MA, USA, Cat# SCM133) and were centrifuged at 300 × g for 5 min. The cell pellet was gently resuspended and transferred to low attachment plates with DMEM/F12 + GlutaMAX supplemented with Knock-Out Serum Replacement (Gibco/Fisher, Cat# 10-828-028), non-essential ammino acids (NEAA) (Gibco/Fisher Scientific, Cat# 11-140-050), 2-mercaptoethanol (Gibco/Fisher Scientific, Cat# 21-010-046) and penicillin-streptomycin (Gibco/Fisher Scientific, Cat# 15-140-122). 10 μM each of ROCK inhibitor Y-27632 and dual SMAD inhibitors SB431542 (Selleck Chemical, Cat# 50-797-0) and dorsomorphin (Selleck Chem, Cat# S7306) were also added to the media. After two days, the media were replaced with fresh media supplemented with dual SMAD inhibitors. The media were replaced daily, until day 6 when culture media were changed to neuronal media containing Neurobasal-A (Gibco/Fisher, Cat# 10-888-022) supplemented with 2% Gibco B-27 serum-free supplement without vitamin A (Fisher Scientific, Cat# 12-587-010), 1% GlutaMAX Supplement (Gibco/Fisher, Cat# 35-050-061), and 1% penicillin/streptomycin (Gibco/Fisher Scientific, Cat# 15-140-122). HCSs were grown in media supplemented with 20 ng/mL of bFGF (Shenandoah Biotechnologies/VWR, Cat# 10821-972) and epidermal growth factor (EGF) (Shenandoah Biotechnologies/VWR, Cat# 10787-462) from days 6–25, and 20 ng/mL of Brain Derived Neurotropic Factor (Shenandoah Biotechnologies/VWR, Cat# 10788-816) and Neurotrophin-3 (Shenandoah Biotechnologies, Cat# 800-06-100ug) from days 26–42 with media replaced every other day. Between days 25–42, hCSs were moved from 6 well culture dishes to 24 well culture dishes with 1 or 2 spheroids per well. From day 43 onward, hCSs were maintained in neuronal media without added factors, and media were replaced every four days.

### RT-PCR validation of VPA DEGs

RNA from control and VPA treated hCSs was collected and purified as described previously. RNA quality and concentration was determined by the NanoDrop 1000 Spectrophotometer (Thermo Fisher Scientific). Stranded cDNA was then prepared using iScript^TM^ cDNA Synthesis Kit (Bio-Rad Laboratories, Inc, USA, Cat#1 70-8891) and the Programmable Thermal Controller, PTC-100 (MJ Research, Inc) following the protocol provided with the kit. The cDNA samples were then prepared for Real-Time Polymerase Chain Reaction (RT-PCR) by using SsoAdvanced Universal SYBR Green Supermix (Bio-Rad Laboratories, Inc, Cat# 172-5271), forward and reverse primers, and cDNA according to the kit protocol. Gene expression was quantified using the QuantStudio3^TM^ Real-Time PCR System (Applied Biosystems/ThermoFisher Scientific, Cat# A28137).

### Primary mouse neuron culture

For primary mouse neuron cultures, #1.5 acid-washed glass coverslips were added to a 12-well culture plate, coated with 1 ml per well of a 50 μg Poly-D-Lysine (Gibco, Cat# A3890401) and 2 μg/mL laminin solution in sterile diH_2_O, put in a 37 °C water-jacketed incubator for 48–72 h, rinsed twice with sterile diH_2_O, and allowed to dry prior to cell plating. On the day of plating, cortices were carefully dissected from embryonic day (E)18.5 mouse brains and dissociated following an adapted protocol described in Bunner et al. [112]. Briefly, for dissociation, cortices were transferred into a 50 mL sterile centrifuge tube containing 10 mL of ice-cold Dissociation medium with Kynurenic acid and MgCl_2_ (DM/Ky.Mg) and washed with ice-cold DM/Ky.Mg 2–3 times followed by 1–2 washes in ice-cold 1× HBSS without Ca^2+^, Mg (Fisher Scientific, Cat# SH3058801). Cortices were then dissociated using the Primary Neuron Isolation Kit and triturated in growth media with serum and then transferred to a solution of warm Opti-ΜΕΜ | Reduced Serum Medium, with glucose (Gibco/Fisher Scientific, Cat# 31-985-062) for centrifugation. Pelleted cells were resuspended in fresh growth media with serum, counted and plated at a density of 500,000 cells/well directly onto glass coverslips. After 2 days in vitro (DIV), the cultures were changed to Prenatal Mouse Neuron Media (PMNM) composed of Neurobasal media (Gibco/Fisher Scientific, Cat# 21-103-049) supplemented with B-27 Plus (Fisher Scientific, Cat# A3582801), GlutaMAX Supplement, and penicillin/streptomycin. The cells were given a ½ media change of PMNM every other day until 14 DIV.

### Immunostaining of hCSs and mCNs

On day 89, all hCSs received fresh neuronal media and any hCSs in the SFN pre-treatment group were dosed with SFN. After 24 h, media were changed once more with the addition of VPA, SFN, VPA and SFN, or DMSO vehicle for control. Following 24 h of exposure, hCSs were washed once with 1× PBS and fixed in 4% PFA initially for 15 min at room temperature followed by 24 h at 4 °C. HCSs were then rinsed with 1× PBS and placed in 30% sucrose for 24 h at 4 °C. Samples were then embedded in Tissue-Tek® O.C.T. Compound (Sakura Finetek USA Inc., Torrance, CA, USA Cat# 4583) overnight, flash frozen, and 10 μm thick cryosections were cut and mounted on microscope slides. Cryosections were warmed to room temperature and permeabilized with 0.2% TritonX-100 in 1× PBS before immunostaining. 5% NGS in 1× PBS was used to block for 30 min at room temperature. Samples were incubated with primary antibodies diluted in 2% NGS in 1× PBS at 4 °C overnight. Continuing at room temperature, slides were rinsed three times with 1× PBS prior to the addition of secondary antibodies diluted in 2% NGS in 1× PBS and incubation at room temperature in the dark for 1 h. Slides were washed twice with 1× PBS and then incubated for 10 min with DAPI diluted 1:1000 in 1× PBS. Slides were subsequently washed twice in 1× PBS and once in deionized water (diH_2_O) before mounting with Fluoro-gel mounting medium (Electron Microscopy Sciences, Hatfield, PA, USA Cat# 1798510).

On 14 DIV, mCNs in the SFN pre-treatment group were dosed with SFN along with the scheduled half media change. After 24 h, half of the media were changed once more with the addition of VPA, SFN, VPA and SFN, or DMSO vehicle for control. Following 24 h of exposure, hCNs were washed once with 1× PBS and fixed in 4% PFA initially for 10 min on ice followed by 10 min with ice cold methanol for 10 min. The fixed cells we washed with 1× TBS three times for five minutes prior to being blocked with 5% NGS and processed for confocal microscopy.

### Confocal microscopy

#### Synaptic stains

HCS sections and fixed mCNs were immunostained with primary antibodies against synaptic markers VGLUT-1 (Synaptic Systems, Göttingen, Germany, Guinea Pig Polyclonal, Cat# 135 304, RRID:AB_2621384, dilution 1:1000) and PSD-95 (Santa Cruz Biotechnology, Dallas, TX, USA, Mouse Monoclonal, Cat# sc-32291, RRID:AB_628113, dilution: 1:50) and secondary Anti-Guinea Pig Alexa Fluor 647 (Invitrogen/Fisher Scientific, Goat Polyclonal, Cat# A-21450, RRID:AB_141882, dilution: 1:500) and Anti-Mouse Alexa Fluor 568 (Invitrogen/Fisher Scientific, Goat Polyclonal, Cat# A-11004, RRID:AB_2534072, dilution: 1:500) antibodies. Cellular adhesions marked by endogenous paxillin-GFP were labeled with anti-GFP primary antibodies (Life Technologies, Carlsbad, CA, USA, Cat# A-11122, RRID:AB_221569, dilution: 1:500) and Anti-Rabbit Alexa Fluor 488 secondary antibodies (Invitrogen/Fisher Scientific, Goat Polyclonal, Cat# A-11008, RRID:AB_143165, dilution: 1:500) in the m-EGFP-tagged Paxillin (PXN) WTC iPSC line, while the actin cytoskeleton was labeled with Acti-stain 488 (Cytoskeleton, Inc., Denver, CO, USA, Cat# PHDG1-A) in the TagBFP-tagged d-Cas9-KRAB WTC iPSC lines. Stained hCSs were imaged on ZEISS LSM 700 confocal microscope with the 40×/1.4 Oil Plan Apochromat objective using the 639, 555, 488, and 405 channels. Z-stacked images were acquired from three areas around the edge of each hCS with 10 z-planes taken over a total depth of 3.125 μm. The open-source ImageJ platform Fiji [109] was used for quantification and analysis beginning with the generation of maximum intensity projections of each z-stack. Each image was converted to an 8-bit tiff file and all samples within the same experiment were held to the same threshold restriction. For our analyses, area measurements of VGLUT-1 and PSD-95 markers and colocalized synapse mask in hCSs were normalized the to the total area of either paxillin-GFP in the m-EGFP-tagged Paxillin (PXN) WTC iPSC line (S. Fig. 5A) or the actin cytoskeleton in the TagBFP-tagged d-Cas9-KRAB WTC iPSC lines (Fig. 5A). Before comparison across treatment groups, we confirmed neither VPA, SFN, nor co-treatment of VPA and SFN produced significant differences to the area of either cellular molecule used to normalize our synaptic markers (S. Fig. 5B). N = 18 micrographs for control, VPA, SFN, and VPA + SFN (3 each from 6 independent experiments analyzed per treatment group), N = 9 for SFN pre-treatment + VPA (3 each from 3 independent experiments analyzed per treatment group). For analysis of mCNs, area measurements of VGLUT-1 and PSD-95 markers and colocalized synapse mask were normalized the number of DAPI. N = 9 micrographs for each treatment (3 each from 3 independent experiments analyzed per treatment group).

#### pNRF2 quantification

HCS sections were immunostained with primary anti-pNRF2 antibodies (Abcam, Cambridge, UK, phospho-Ser40, Rabbit monoclonal antibody, Cat# ab76026, RRID: AB_1524049, Dilution 1:200) and Anti-Rabbit Alexa Fluor 647 antibodies (Invitrogen/Fisher Scientific, Goat Polyclonal, Cat# A-21245, RRID: AB_2535813, Dilution: 1:500) as previously described. After immunostaining, hCSs were imaged on ZEISS LSM 800 confocal microscope with the 40×/1.4 Oil Plan Apochromat objective using the 639 and 405 channels. For each channel, we obtained z-stacked images as previously described over 2.52 μm. These slices were summed in Fiji and converted to 8-bits for analysis. To count individual nuclei, the DAPI channel underwent gaussian blur correction with sigma = 3, default auto thresholding to remove background, and conversion to a binary mask. The watershed function on Fiji was used to separate merged nuclei and used particle analysis to create outlines of each nucleus and add each area to the ROI manager. The pNRF2 channel was held to a consistent threshold within each replicate and the pNRF2 area fraction for each nucleus was recorded using the ROI manager. For our analysis, we defined pNRF2 positive nuclei as an area fraction >10%. In the hCS, this threshold of 10% overlap allows for distinguishing between true signal and overlap of tightly packed nuclei. N = 18 micrographs for control, VPA, SFN, and VPA + SFN (3 each from 6 independent experiments analyzed per treatment group), N = 9 for SFN pre-treatment + VPA (3 each from 3 independent experiments analyzed per treatment group).

### Microelectrode array (MEA) analysis

For hCS cultures, 24-well CytoView MEA plates (Axion Biosystems, Atlanta, GA, USA, Cat# M384-tMEA-24W) were prepared 48 h prior to cell plating as follows: wells were first coated with a solution of 0.1% polyethyleneimine (Sigma-Aldrich, Cat# P3143) in borate buffer (pH 8.4) incubated for 1 h at 37 °C, rinsed four times with sterile diH_2_O and dried over night at room temperature. The following day, 5 μg/mL mouse laminin (VWR, Cat# 47743-734) in 1× PBS was added to each well and left at room temperature overnight. Prior to plating, the laminin was replaced with Hank’s Balanced Salt Solution (HBSS) and incubated at 37 °C for one hour. 90-day old hCSs were dissociated using Primary Neuron Isolation Enzyme with papain (Pierce/Thermo Fisher Scientific/Fisher Scientific, Cat# PI88285) and plated at 250,000 cells/well onto the coated MEA plates. Cultures were given Neurobasal-A medium supplemented with B-27 Plus without vitamin A (Fisher Scientific, Cat# A3582801), GlutaMAX Supplement, and penicillin/streptomycin. Cultures were allowed to reform connections and establish consistent activity over 30 days. MEA experiments were done in triplicate, resulting in 8–10 wells per condition for statical analysis.

For primary mouse neuron cultures, 24-well MEA plates were coated with a 50 μg Poly-D-Lysine (Gibco, Cat# A3890401) and 2 μg/mL laminin solution in sterile diH_2_O overnight and rinsed twice with sterile diH_2_O and allowed to dry prior to cell plating. On the day of plating, cortices were carefully dissected from embryonic day (E)18.5 mouse brains and dissociated as above. Pelleted cells were resuspended in fresh growth media with serum, counted and plated directly onto MEA electrodes in a 5 μL drop containing 10,000 cells. Cells were allowed to adhere to the electrode for 30 min at 37 °C before the addition of 1 mL of fresh growth media. After 2 days in vitro (DIV), the cultures were changed to Prenatal Mouse Neuron Media composed of Neurobasal media (Gibco/Fisher Scientific, Cat# 21-103-049) supplemented with B-27 Plus (Fisher Scientific, Cat# A3582801), GlutaMAX Supplement, and penicillin/streptomycin. Cultures were allowed to reform connections and establish consistent activity over 14 DIV. MEA experiments were done in triplicate, resulting in 17–18 wells per condition.

The Axion Biosystems Maestro Edge Multielectrode Array System and AxIS Navigator software (Axion Biosystems, Version 3.7.1.16) were used to record the spontaneous action potentials of the 2D cultures. Twenty-four hours before each experiment, media were replaced with 750 μL fresh media containing 1 mM HEPES (Gibco/Fisher Scientific, Cat# 15-630-080). On the day of each experiment, baseline activity of the neural networks was recorded prior to the addition of 250 μL of 4× concentrated medias obtaining in a final concentration of 500 μM VPA, 0.1 μM SFN, or 500 μL VPA and 0.1 μM SFN together as used in prior experiments. The cultures were exposed to their respective drug or control for 24 h, and we obtained records of the neural activity at 3-, 6- and 24-h time points. After conclusion of the last reading, the cells were washed with warm 1× PBS and provided with 1 mL fresh media supplemented with HEPES. An additional recording was taken after a 24-h washout for both hCSs and mCNs. All recorded electrical activity underwent DC offset filtering and Butterworth band-pass filtering with 0.1 Hz and 5 kHz cutoffs respectively prior to spike detection. The spike detector was set to record any electrical event with a peak voltage ≥6 times the standard deviation of the estimated signal from background noise. We used the Neural Metric Tool (Axion Biosystems, Version 4.0.5) for post-processing and exporting neural metrics associated with each recording. In post-processing, bursts were defined as 5 or more spikes with ≤100 ms separating each spike. Synchrony was calculated as the area under the normalized cross-correlation, which measures well-wide pooled inter-electrode cross-correlation and normalizes to the auto-correlations within individual electrode.

### Additional reagents

R,S-Sulforaphane was purchased from LKT Laboratories, Inc. (Saint Paul, MN, USA, Cat# S8047). Valproic Acid Sodium Salt was purchased from Sigma-Aldrich (Cat# P4543). On the day of experimentation, neat SFN was dissolved in DMSO solvent and then diluted to the appropriate concentration in cell culture media. SFN was independently tested by HPLC analysis conducted by Scout Scientific LLC (Greenville, NC USA) to confirm manufacturer purity and reanalyzed annually or when a new aliquot was purchased in conformity with NCCIH Natural Product Integrity Policy. Using HPLC, we found that SFN storage in DMSO resulted in degradation and therefore was stored as a neat solution.

### Statistical analyses

Statistical analyses were performed using GraphPad Prism 10 Software (GraphPad Software, LLC, Version 10.1.1 (270)). Individual statistical methods used, and sample sizes are described in figure legends. Datasets were first tested for normality and equal variance using Shapiro-Wilks test and Bartlett’s test, respectively. For data with three or more groups, one-way ANOVA followed by Tukey’s multiple comparisons test when p < 0.05 was used for datasets where all groups passed the normality and variance tests. Non-parametric data were analyzed using one-way ANOVA on ranks (Kruskal-Wallis test) with Dunn’s multiple comparisons test when p < 0.05. For VPA dose response analysis, uncorrected Fisher’s Least Significant Difference (LSD) test was used in place of multiple comparisons after ANOVA to prevent type II error arising from high variance [113]. For comparison of two groups Mann-Whitney test was used.

## Supplementary information

Supplementary Figure Legends

S. Fig. 1

S. Fig. 2

S. Fig. 3

S. Fig. 4

S. Fig. 5

S. Table 1

S. Table 2

S. Table 3

## Data Availability

Raw data sets will be made available upon reasonable request to the corresponding author.
