# Supplementary material for: Sulforaphane protects developing neural networks from VPA-induced synaptic alterations
Source: Mol Psychiatry. 2025 Apr 2;30(9):3868–84. doi: 10.1038/s41380-025-02967-5 (PMC12339368; doi:10.1038/s41380-025-02967-5)
Supplement: Supplementary file 1 — Supplementary Figure Legends [file 41380_2025_2967_MOESM1_ESM.docx]

**Supplementary Figure Legends:**

Supplementary Figure 1:

**(A)** Quantification of pNRF2 positive nuclei hNPCs treated with SFN compared to control (Data represented as Mean ± SEM; N = 3; data analyzed using Kruskal-Wallis test (p=0.0026) with Dunn's multiple comparisons test; **p<0.01 and *p<0.05). **(B)** Percent distribution of pNRF2 positive and negative nuclei for all hNPCs. Positive nuclei correlate to greater than 10% overlap with DAPI divided into groups with 10-25%, 25-50%, 50-75%, or 75-100% overlap. **(C)** Representative confocal images of NRF2 with treatments of 0.1 μm SFN, 1.0 μm SFN, 10 μm SFN. White scale bar represents 100 μm. **(D)** Quantified NRF2 area normalized to DAPI was compared to control group, represented as fold change from control (N = 3 experiments; analyzed using Kruskal-Wallis test (p=0.0091) with Dunn's multiple comparisons test). **(E)** Representative widefield images for quantified data in Fig. 1D of CellROX™ Deep Red in hNPCs treated with 2.5 μM menadione. 500 μM VPA and Control images are duplicated from Fig. 1D for visual comparison of signal. White scale bar represents 200 μm.

Supplementary Figure 2:

**(A)** Representative widefield images of Ki67 and DAPI in hNPCs treated with vehicle control, 500 μM VPA, 0.1 μM SFN, or the combination of 0.1 μM SFN and 500 μM VPA. White scale bar represents 200 μM. **(B)** Quantification of Ki67 positive nuclei in treated hNPCs compared to control (Data represented as Mean ± SEM; N = 3; data analyzed using Kruskal-Wallis test (p=0.0026) with Dunn's multiple comparisons test; **p<0.01 and *p<0.05).

Supplementary Figure 3:

Quantification of pNRF2 area in each treatment group compared to control normalized to DAPI area (Data represented as Mean ± SEM; N = 18 for control, VPA, SFN, and VPA + SFN, N = 9 for SFN pre-treatment + VPA; data analyzed using Kruskal-Wallis test (p=0.0026) with Dunn's multiple comparisons test; **p<0.01 and *p<0.05).

Supplementary Figure 4:

RT-PCR results for 3 up regulated and 3 down regulated DEGs selected from RNA-seq experiment. VPA treated human cortical spheroids (hCSs) transcript abundance was normalized to control hCSs, 18s transcript abundance was used an internal control. Data represented as mean ± SEM; N = 4-5 hCSs.

Supplementary Figure 5:

**(A)** Representative confocal images of fluorescently labeled endogenous PXN-GFP (white) and colocalized synapse mask (yellow) after 24-hour treatment of vehicle control, 500 μM VPA, 0.1 μM SFN, or 500 μM VPA and 0.1 μM SFN. White scale bar represents 50 μm. **(B)** Quantified actin area normalized to total captured hCS area. **(C)** Quantified PXN area normalized to total captured hCS area. **(B and C)** Data were analyzed using Kruskal-Wallis test, both p>0.05.
