# Supplementary figures and images for: Sulforaphane protects developing neural networks from VPA-induced synaptic alterations
Source: Mol Psychiatry. 2025 Apr 2;30(9):3868–84. doi: 10.1038/s41380-025-02967-5 (PMC12339368; doi:10.1038/s41380-025-02967-5)

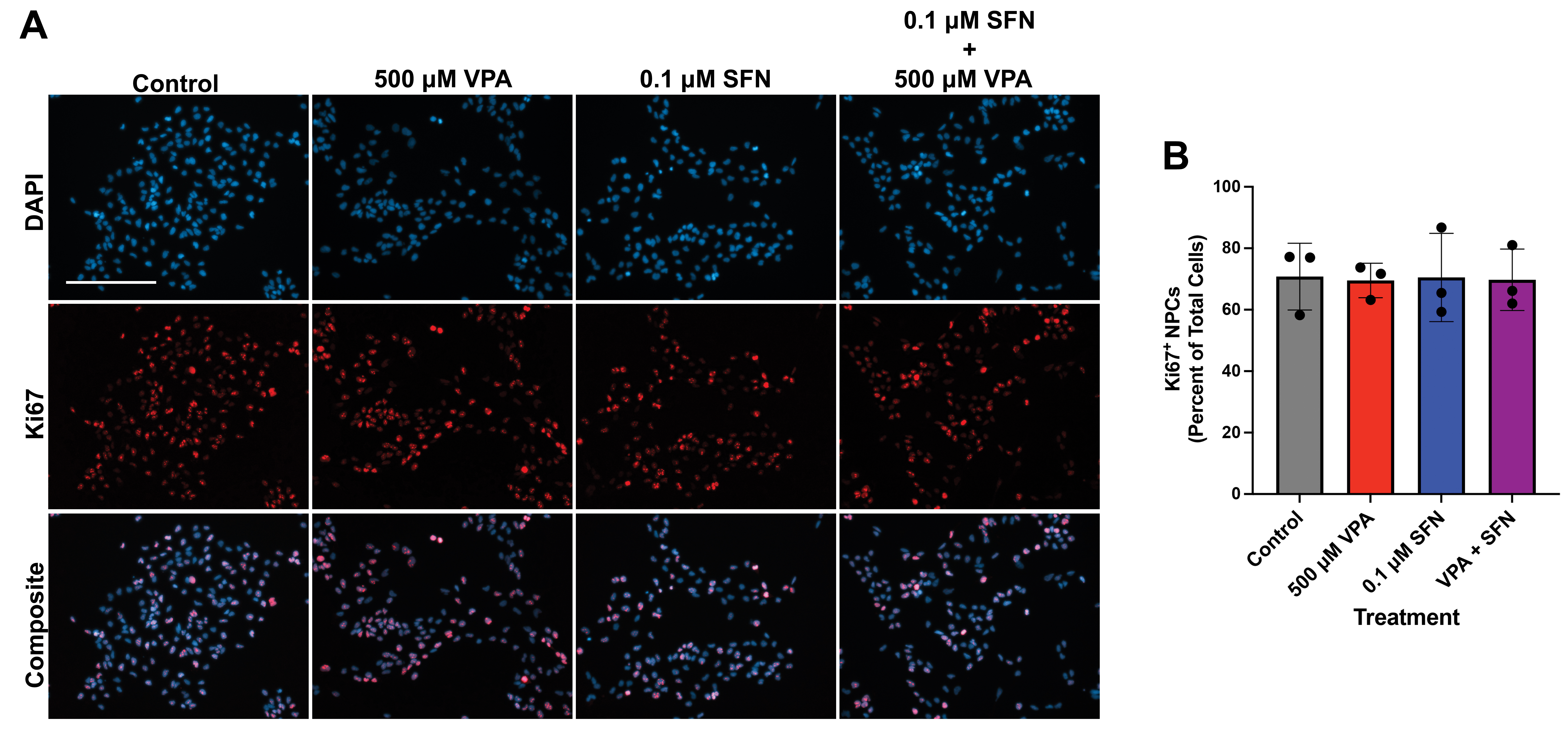

Supplement: Supplementary file 3 — S. Fig. 2 [file 41380_2025_2967_MOESM3_ESM.jpg]

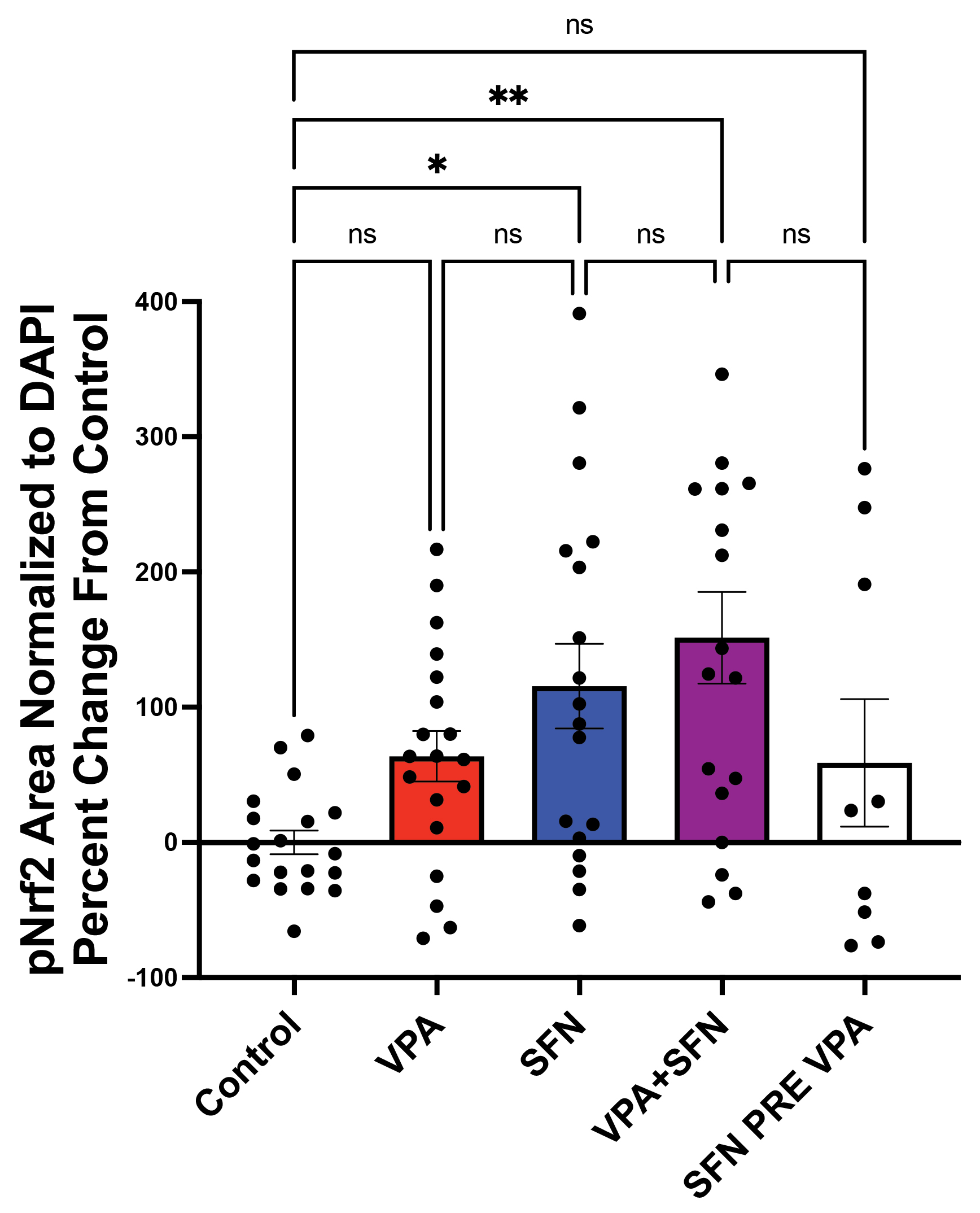

Supplement: Supplementary file 4 — S. Fig. 3 [file 41380_2025_2967_MOESM4_ESM.jpg]

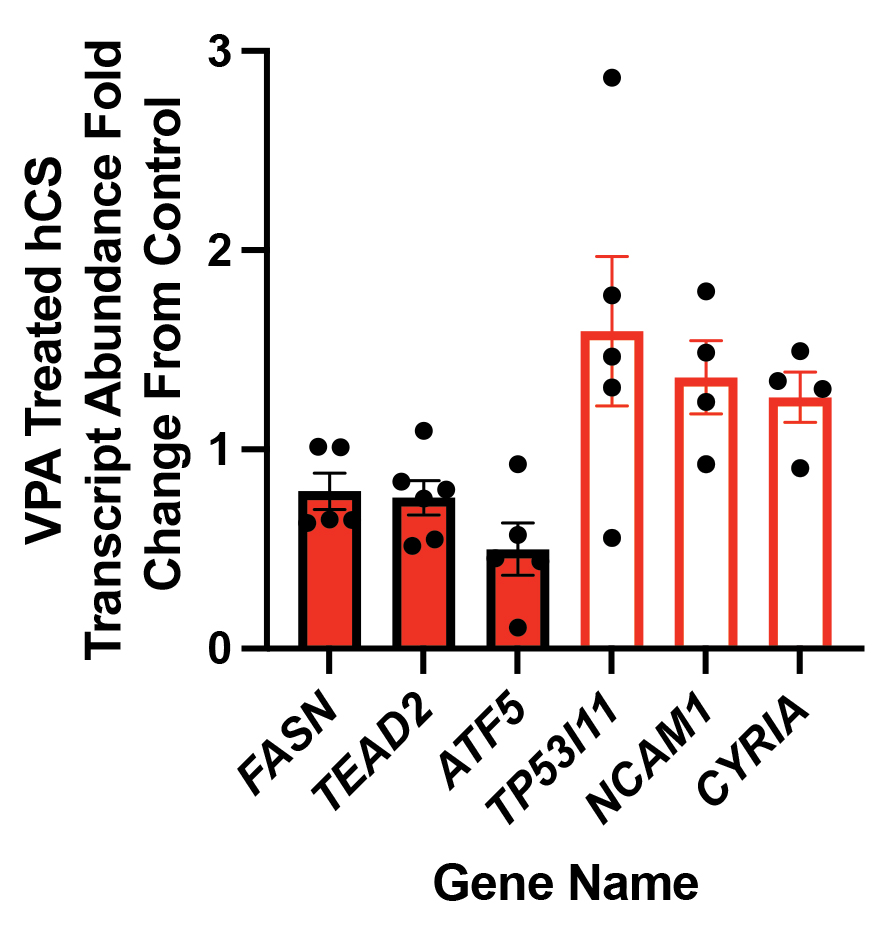

Supplement: Supplementary file 5 — S. Fig. 4 [file 41380_2025_2967_MOESM5_ESM.jpg]

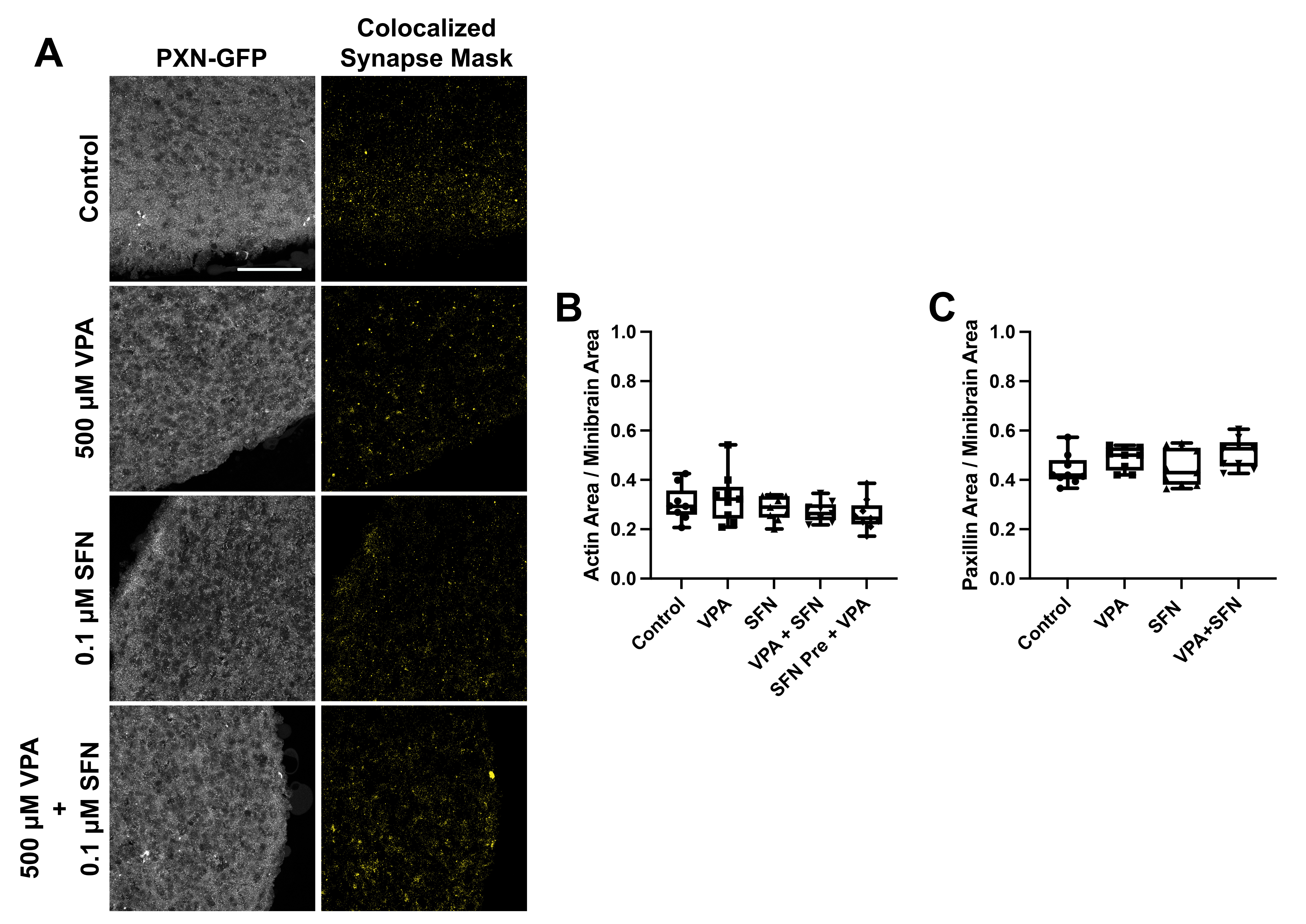

Supplement: Supplementary file 6 — S. Fig. 5 [file 41380_2025_2967_MOESM6_ESM.jpg]

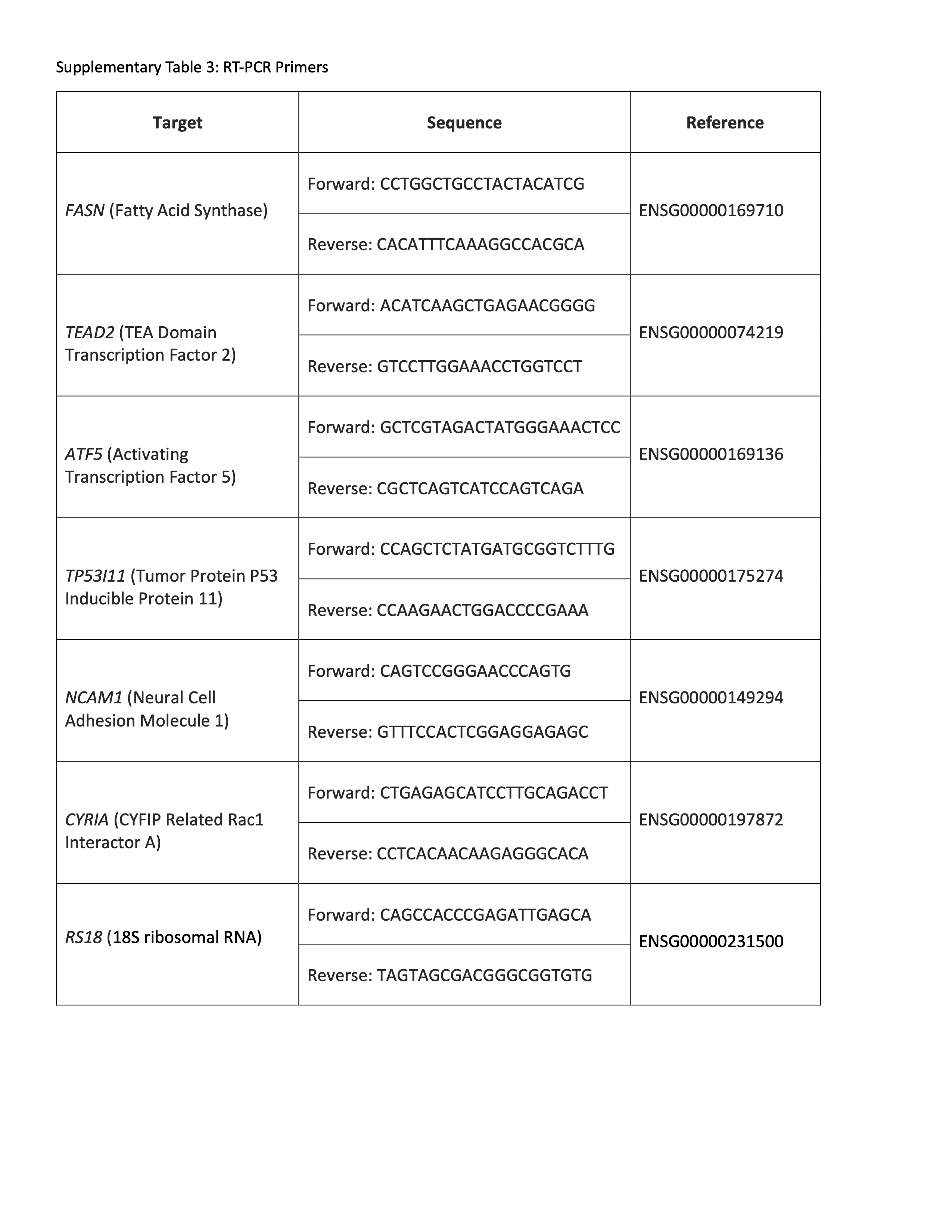

Supplement: Supplementary file 9 — S. Table 3 [file 41380_2025_2967_MOESM9_ESM.tif]
